# CD32^+^CD4^+^ T Cells Are Highly Enriched for HIV DNA and Can Support Transcriptional Latency

**DOI:** 10.1016/j.celrep.2020.01.071

**Published:** 2020-02-18

**Authors:** Gilles Darcis, Neeltje A. Kootstra, Berend Hooibrink, Thijs van Montfort, Irma Maurer, Kevin Groen, Suzanne Jurriaans, Margreet Bakker, Carine van Lint, Ben Berkhout, Alexander O. Pasternak

**Affiliations:** 1 Laboratory of Experimental Virology, Department of Medical Microbiology, Amsterdam UMC, University of Amsterdam, Amsterdam, the Netherlands; 2 Infectious Diseases Department, Liège University Hospital, Liège, Belgium; 3 Laboratory of Viral Immune Pathogenesis, Department of Experimental Immunology, Amsterdam UMC, University of Amsterdam, Amsterdam, the Netherlands; 4 Department of Cell Biology, Amsterdam UMC, University of Amsterdam, Amsterdam, the Netherlands; 5 Laboratory of Clinical Virology, Department of Medical Microbiology, Amsterdam UMC, University of Amsterdam, Amsterdam, the Netherlands; 6 Service of Molecular Virology, Département de Biologie Moléculaire (DBM), Université Libre de Bruxelles (ULB), Gosselies, Belgium; 7 Lead Contact

## Abstract

The HIV latent reservoir forms the major hurdle to an HIV cure. The discovery of CD32 as marker of this reservoir has aroused much interest, but subsequent reports have challenged this finding. Here, we observe a positive correlation between the percentages of CD32^+^ cells among CD4^+^ T cells of aviremic cART-treated, HIV-infected individuals and their HIV DNA loads in peripheral blood. Moreover, optimization of the CD32^+^CD4^+^ T cell purification protocol reveals prominent enrichment for HIV DNA (mean, 292-fold) in these cells. However, no enrichment for HIV RNA is observed in CD32^+^CD4^+^ cells, yielding significantly reduced HIV RNA/DNA ratios. Furthermore, HIV proviruses in CD32^+^CD4^+^ cells can be reactivated *ex vivo* to produce virus, strongly suggesting that these cells support HIV transcriptional latency. Our results underscore the importance of isolating pure, bona fide CD32^+^CD4^+^ T cells for future studies and indicate that CD32 remains a promising candidate marker of the HIV reservoir.

## In Brief

CD32a was recently proposed to mark the HIV reservoir, but this finding was subsequently challenged. By using a sequential cell-sorting protocol to purify bona fide CD32^+^CD4^+^ cells, Darcis et al. demonstrate HIV DNA enrichment and ex vivo reactivation-mediated virus production in these cells, reinforcing CD32 as an HIV reservoir marker.

## Graphical Abstract

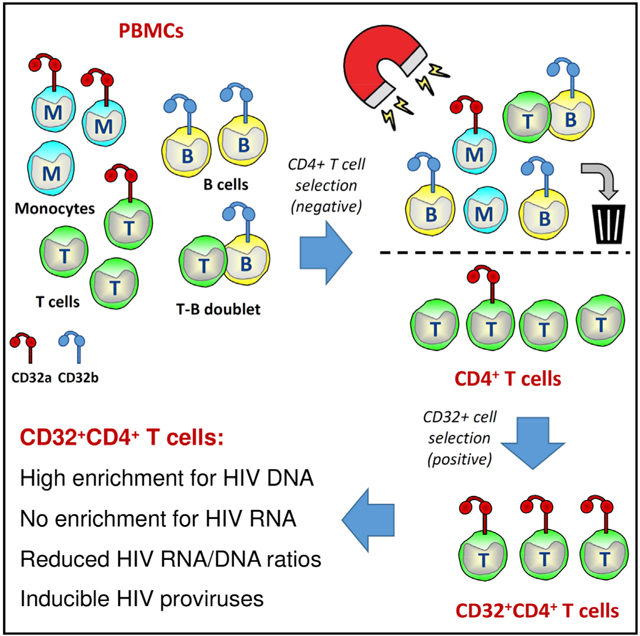

## INTRODUCTION

Combination antiretroviral therapy (cART) allows clinicians to successfully manage most HIV-infected individuals, to prevent the development of AIDS, and to considerably reduce the risk of virus transmission. Unfortunately, cART is not curative, and treatment interruption usually leads to a fast viral rebound (Chun et al., 1995, 1997, 2000; Davey et al., 1999; Finzi et al., 1999; Siliciano et al., 2003). Treatment has to be taken for life, and finding a cure for HIV remains an extremely important, but thus far unattainable, goal. The source of the viral rebound after therapy is stopped is the latent HIV reservoir, which is considered the major hurdle to an HIV cure. The viral reservoir is typically defined as a cell type or anatomical site where replication-competent virus can persist for a prolonged period (Darcis et al., 2017, 2018; Pasternak and Berkhout, 2016). However, defective proviruses have been proposed to play a role in HIV pathogenesis through the production of viral proteins that cause chronic immune activation (Douek, 2003; Imamichi et al., 2016; Pollack et al., 2017). Therefore, the definition of the viral reservoir could be extended to all infected cells, including those infected with defective proviruses (Avettand-Fènoël et al., 2016). In line with this, total, but not intact, HIV DNA copy numbers have been demonstrated to predict post-treatment HIV control (Sharaf et al., 2018; Williams et al., 2014). The main reservoir is thought to consist of long-lived resting memory CD4^+^ T cells (Finzi et al., 1997). HIV can persist during cART in central, transitional, and effector memory CD4^+^ T cells, in addition to naive CD4^+^ T cells (Chomont et al., 2009; Khoury et al., 2016; Wightman et al., 2010). Among memory CD4^+^ T cells, effector memory cells contain the larger proportion of intact HIV genomes (Hiener et al., 2017). CD4^+^ T memory stem cells stand out as another cell population in which long-term HIV persistence is particularly evident, likely because of their superior ability for self-renewal, resistance to apoptosis, and extended lifespan (Buzon et al., 2014; Gattinoni et al., 2011). Lymph node follicular helper T cells, as well as their blood-circulating counterpart, represent yet another cellular location for persisting virus during cART (Banga et al., 2016, 2018).

Although significant progress has been made in our understanding of HIV biology and pathogenesis, the composition and dynamics of the viral reservoir and the mechanisms of HIV persistence remain largely ill-defined. The HIV cure field invested heavily to sort out the “right” HIV reservoir cells from the plethora of cells in an infected individual (Pasternak and Berkhout, 2016), but the absence of an easy marker for latently infected cells poses a major block to better understanding of the HIV reservoir. Several markers of latently infected cells recently have been suggested. Fromentin et al. (2016) showed that immune checkpoint molecules (ICs) PD-1, TIGIT, and LAG-3 were positively associated with the frequency of CD4^+^ T cells harboring HIV DNA: memory CD4^+^ T cells co-expressing those three markers were enriched for HIV up to 10 times compared with total CD4^+^ T cells. ICs may favor HIV latency during cART through their ability to inhibit T cell activation. Iglesias-Ussel et al. (2013) demonstrated that CD4^+^ T cells expressing high surface levels of CD2 harbored higher HIV DNA copy numbers (range, 3- to 10.8-fold) compared with total CD4^+^ T cells. High CD2-expressing cells may be infected more readily by direct binding of the HIV envelope to CD2 or by enhanced interaction with antigen-presenting cells (APCs), which may also boost virus transmission. However, the level of HIV DNA enrichment observed in these two studies is quite modest. More recently, Hogan et al. (2018) showed that CD4^+^ T cells expressing CD30, a member of the tumor necrosis factor receptor superfamily, are enriched for cell-associated (CA) HIV RNA. However, this enrichment was not observed in all studied individuals, and CD30^+^CD4^+^ T cells were not significantly enriched for HIV DNA.

Recently, CD32a was proposed as marker of the CD4^+^ T cell HIV reservoir (Descours et al., 2017). CD32a is the low-affinity receptor for the immunoglobulin G Fc fragment that is highly expressed on myeloid cells and expressed on a small subset of T cells (Hogarth and Pietersz, 2012; Holgado et al., 2018). In contrast to the limited fold enrichment observed for other candidate markers, extremely high enrichment for HIV DNA of ~1,000-fold was observed in CD4^+^ T cells with high CD32a expression compared with CD32^−^CD4^+^ T cells (Descours et al., 2017). If confirmed, this finding would represent a milestone in our efforts to develop a cure for HIV infection by providing a “handle” for identification and selective targeting of the reservoir (Darcis et al., 2019). However, several reports questioned whether CD32a is a bona fide marker of the HIV latent reservoir (Abdel-Mohsen et al., 2018; Badia et al., 2018; Bertagnolli et al., 2018; Martin et al., 2018; Osuna et al., 2018; Pérez et al., 2018). In particular, these studies could not reproduce the main finding of Descours et al. (2017), because no HIV DNA enrichment in CD32^+^CD4^+^ cells could be observed.

In this report, we aimed to investigate the contribution of CD32^+^CD4^+^ T cells to HIV persistence. We developed a sequential, multiple-round, magnetic bead cell-sorting protocol and quantified HIV DNA and CA unspliced (US) HIV RNA in the CD32^+^CD4^+^ and CD32^−^CD4^+^ T cell fractions (Pasternak and Berkhout, 2018; Pasternak et al., 2013). Using this protocol, we demonstrated that by further purification of the CD32^+^CD4^+^ cells, one can move from no enrichment to prominent enrichment for HIV DNA, but not for CA HIV RNA, in these cells. Despite low basal HIV transcription levels, HIV proviruses in CD32^+^CD4^+^ cells could be reactivated *ex vivo* to produce virus, suggesting that HIV proviruses in these cells are transcriptionally silent but inducible—a hallmark of latent reservoir cells. Combined, our data convincingly demonstrate, by independent means, that CD32^+^CD4^+^ T cells indeed comprise a major component of the HIV reservoir and provide a plausible explanation for the negative results obtained by other groups.

## RESULTS

### Percentage of CD32^+^CD4^+^ T Cells in HIV-Infected Individuals

**Figure 1.**
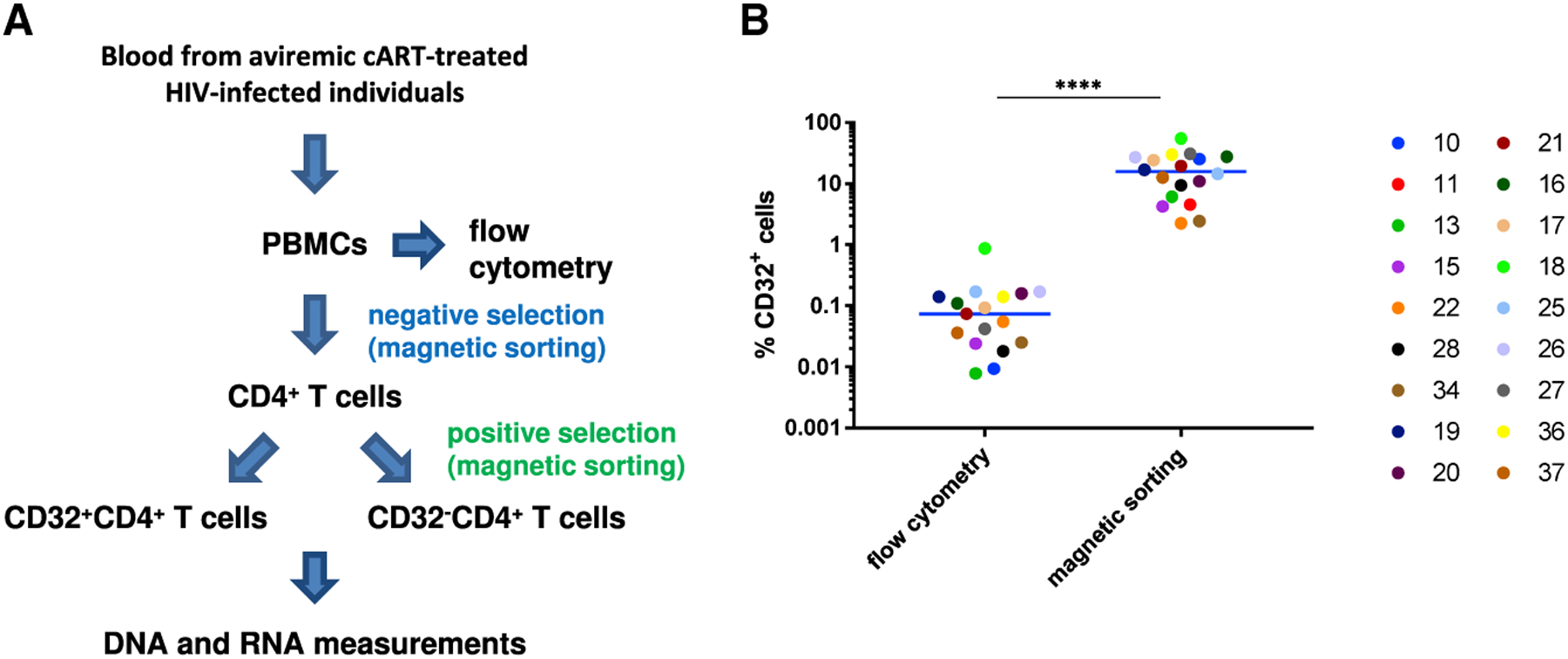
Schematic of the Study (A) Flowchart of cell isolation. Total PBMCs were divided into two parts. The first part was used for flow cytometry. The second part was used for negative magnetic selection for CD4^+^ cells, followed by positive magnetic selection to obtain CD32^+^CD4^+^ and CD32^−^CD4^+^ cell populations that were used for HIV DNA and RNA measurements. (B) Comparison of percentages of CD32^+^ cells among CD4^+^ cells between flow cytometry- and magnetic-sorting-based isolation (n = 18). Wilcoxon test was used to calculate statistical significance. ****p < 0.0001.

We initially studied 18 HIV-infected cART-treated individuals who had been aviremic (plasma viral load < 40 copies/mL) for at least 4 months, with a median of 7 years (set A, Table S1). We first determined the percentage of CD32^+^CD4^+^ T cells by flow cytometry (Figure 1A). To quantify the percentage of CD4^+^ T cells expressing CD32, we defined a specific CD32 gate for each participant using fluorescence minus one (FMO) control (the gating strategy is shown in Figure S1). The anti-CD32 antibody used in our study and the Descours et al. (2017) study does not discriminate between CD32a and CD32b proteins, because their extracellular domains are very similar (Abdel-Mohsen et al., 2018). No specific anti-CD32a antibody is yet available. CD32b is also an Fcγ receptor, but in contrast to CD32a, CD32b is mostly expressed on B cells (Hogarth and Pietersz, 2012). Percentages of CD32^+^ cells among CD4^+^ T cells ranged from 0.008% to 0.87% with a median of 0.074% (Figure 1B, left panel), which is somewhat higher than the percentages reported by Descours et al. (2017).

### CD32^+^CD4^+^ T Cells Co-express HLA-DR, TIGIT, and LAG-3

**Figure 2.**
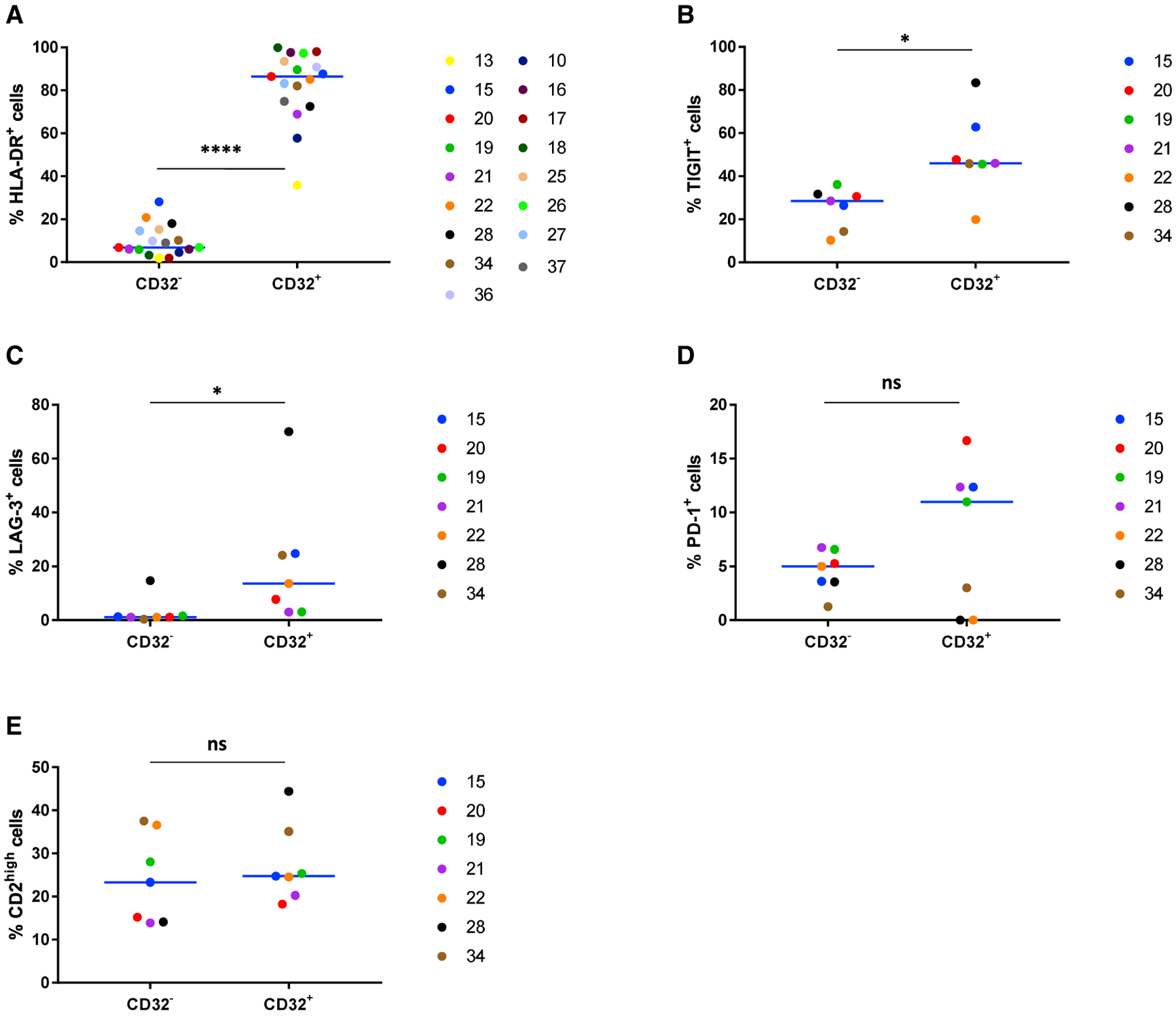
CD32^+^CD4^+^ T Cells Are Enriched for HLA-DR, TIGIT, and LAG-3 Expression (A–E) Expression of HLA-DR (n = 17) (A), TIGIT (n = 7) (B), LAG-3 (n = 7) (C), PD-1 (n = 7) (D), and CD2 (n = 7) (E) was measured by multi-parametric flow cytometry on CD32^−^CD4^+^ and CD32^+^CD4^+^ T cells isolated from HIV-infected individuals. Wilcoxon tests were used to calculate statistical significance. ****p < 0.0001, *0.01 < p < 0.05; ns, not significant.

To study the activation level of CD4^+^ T cells expressing CD32^+^ and to establish whether these cells co-express other markers that were reported to be linked to enrichment for HIV DNA (Fromentin et al., 2016; Iglesias-Ussel et al., 2013), we measured the co-expression of CD32 with HLA-DR, ICs (PD-1, TIGIT, and LAG-3), and CD2 by multi-parametric flow cytometry (Figure 2; Figure S1) on CD4^+^ T cells from HIV-infected individuals. A higher proportion of CD32^+^CD4^+^ T cells expressed the activation marker HLA-DR (median, 86.4%; range, 35.8%–99.8%) compared with CD32^−^CD4^+^ T cells (median, 6.9%; range, 1.8%–28.1%) (p < 0.0001) (Figure 2A). A higher proportion of CD32^+^CD4^+^ T cells expressed TIGIT (46.0%, 19.9%–83.3%, versus 28.5%, 10.3%–36.1%) and LAG-3 (13.6%, 3.0%–70.0%, versus 1.2%, 0.4%–14.7%), compared with CD32^−^CD4^+^ T cells (p = 0.016 for both markers) (Figures 2B and 2C). The median percentage of CD32^+^CD4^+^ cells expressing PD-1 was higher compared with CD32^−^CD4^+^ T cells (11.0%, 0.0%–16.7%, versus 5.0%, 1.3%–6.8%) (Figure 2D), but this difference did not achieve statistical significance (p = 0.22). Finally, the proportions of CD32^+^CD4^+^ and CD32^−^CD4^+^ T cells expressing high levels of CD2 were similar (24.8%, 18.2%–44.4%, versus 23.3%, 13.9%–37.5%) (Figure 2E).

### Percentages of CD32^+^CD4^+^ T Cells Positively Correlate with HIV DNA Levels in PBMC

To determine the level of enrichment for HIV DNA and RNA in CD32^+^ cells, the CD32^+^CD4^+^ and CD32^−^CD4^+^ T cell fractions were isolated from peripheral blood mononuclear cells (PBMCs) by magnetic cell sorting (Figure 1A), and total nucleic acids were extracted from these fractions and total PBMCs. Total HIV DNA and CA HIV US RNA were separately quantified in these extracts by quantitative PCR (qPCR), and their copy numbers were normalized to the cellular DNA or RNA inputs, respectively. To maximize the nucleic acid extraction yields and to prevent artificial DNA or RNA under- or overquantitation when using low cellular inputs, carrier RNA was added to every extraction. This resulted in stable high yields of HIV and cellular DNA and RNA, even from very low cellular inputs, compared with the corresponding extractions without carrier RNA (Figures S2A–S2D). The addition of carrier RNA also resulted in stable HIV DNA and RNA levels when normalized to cellular DNA or RNA, as well as stable HIV RNA/DNA ratios, whereas DNA extractions without carrier RNA resulted in significant overquantitation of HIV DNA relative to cellular DNA and underquantitation of HIV RNA/DNA ratios with low cellular inputs (Figures S2E–S2G). We also tested whether underquantitation of HIV DNA could result from inhibition of the HIV DNA qPCR by cellular DNA and found no inhibition up to 200,000 cell equivalents of DNA per qPCR reaction (Figure S2H). Therefore, in the subsequent measurements, we always included less than 200,000 cell equivalents of DNA in qPCR.

**Figure 3.**
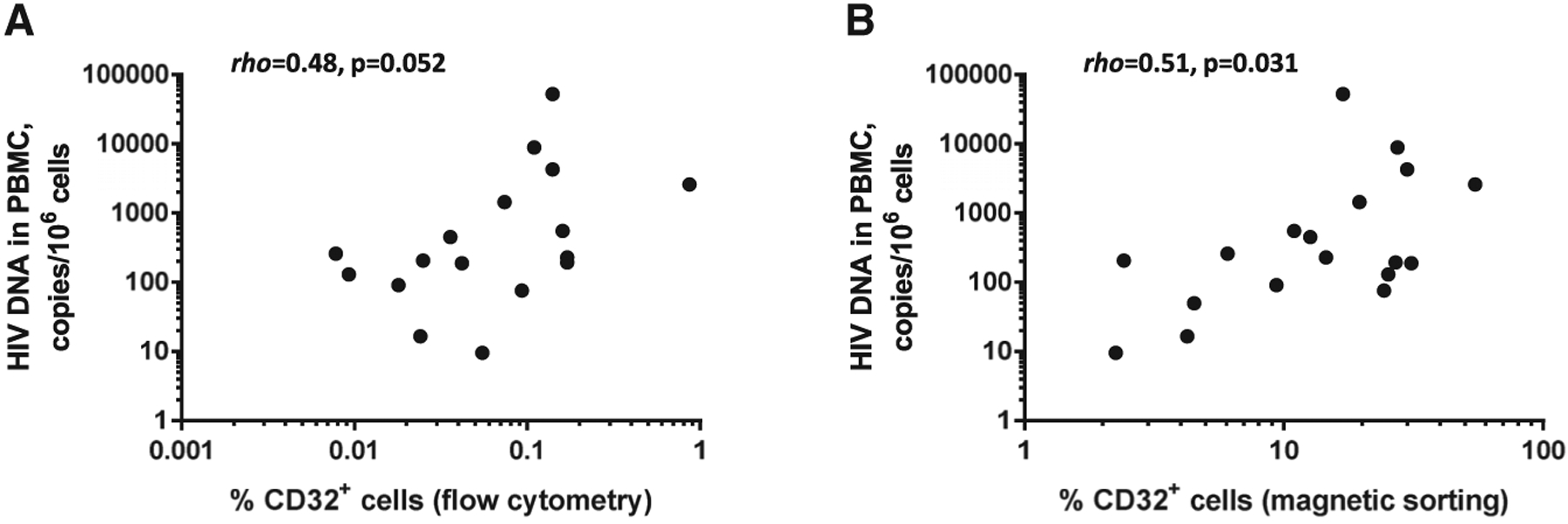
Percentages of CD32^+^ Cells among CD4^+^ T Cells Positively Correlate with HIV DNA in PBMC (A and B) Correlations between percentages of CD32^+^ cells among CD4^+^ T cells and HIV DNA load in PBMC. CD32^+^CD4^+^ cells were isolated by flow cytometry (n = 17) (A) or magnetic sorting (n = 18) (B). Spearman tests were used to calculate statistical significance.

In addition to flow cytometry, percentages of CD32^+^ cells among total CD4^+^ T cells were determined by cellular DNA quantitation in CD32^−^ and CD32^+^ fractions of CD4^+^ cells isolated by magnetic sorting. These percentages were higher than those determined by flow cytometry (p < 0.0001) (Figure 1B), suggesting that a substantial proportion of residual non-CD32^+^CD4^+^ cells is retained in the CD32^+^ fraction after magnetic sorting. Despite this, we observed a positive correlation between the HIV DNA load in PBMCs and the percentage of CD4^+^ T cells that are CD32^+^, the latter measured either by flow cytometry or by magnetic cell sorting (rho = 0.48, p = 0.052, or rho = 0.51, p = 0.031, respectively) (Figure 3).

### Lack of HIV DNA Enrichment in the CD32^+^CD4^+^ Fraction Results from the Presence of Residual Non-T Cells

**Figure 4.**
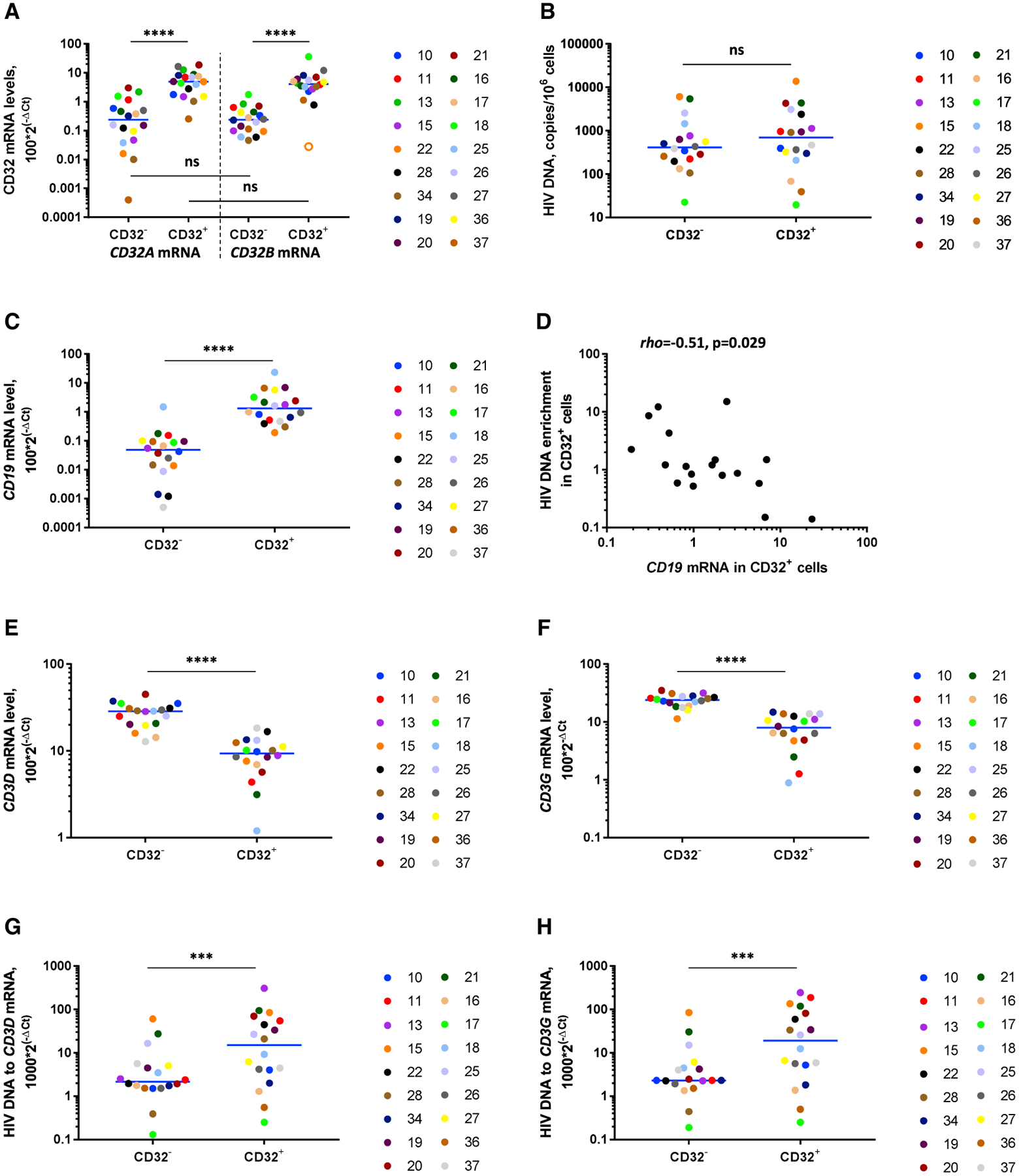
Lack of Enrichment for HIV DNA in the CD32^+^CD4^+^ Fraction Results from the Presence of Residual Non-T Cells in This Fraction (A) *CD32A* and *CD32B* mRNA levels in CD32^+^ and CD32^−^ fractions of CD4^+^ T cells. (B) HIV DNA levels, normalized to the total cellular DNA, in CD32^+^ and CD32^−^ fractions. (C) *CD19* mRNA levels in CD32^+^ and CD32^−^ fractions. (D) Correlation between levels of *CD19* mRNA in the CD32^+^ fraction and HIV DNA enrichment in this fraction. (E and F) *CD3D* (E) and *CD3G* (F) mRNA levels in CD32^+^ and CD32^−^ fractions. (G and H) HIV DNA levels, normalized to *CD3D* (G) and *CD3G* (H) mRNA, in CD32^+^ and CD32^−^ fractions. All mRNA levels were normalized to *GAPDH* mRNA. The open circle depicts an undetectable value, censored to the detection limit. Wilcoxon tests (all panels except D), or a Spearman test (D) were used to calculate statistical significance. ****p < 0.0001, ***0.0001 < p < 0.001; ns, not significant. All panels, n = 18.

First, to confirm the specificity of CD32^+^ cell selection, we measured the *CD32A* and *CD32B* mRNA levels in the CD32^+^CD4^+^ and CD32^−^CD4^+^ fractions. Indeed, prominent differences were observed for both *CD32* mRNAs between these fractions: median enrichment in the CD32^+^ fraction was 24-fold for *CD32A* mRNA and 16-fold for *CD32B* mRNA (both p < 0.0001) (Figure 4A). However, no significant difference was observed between *CD32A* and *CD32B* mRNA levels in either fraction (Figure 4A; Figure S3A).

Next, we measured enrichment for HIV DNA and RNA in CD32^+^CD4^+^ cells compared with CD32^−^CD4^+^ cells. Confirming recent reports (Abdel-Mohsen et al., 2018; Badia et al., 2018; Martin et al., 2018; Osuna et al., 2018; Pérez et al., 2018), but in clear contradiction to the original findings of Descours et al. (2017), CD32^+^ cells were not enriched for either HIV DNA or HIV RNA (Figure 4B; Figure S3B). However, based on the difference between the CD32^+^ cell percentages upon magnetic sorting and those upon flow cytometry (described earlier), and on the notable presence of *CD32B* mRNA, whose protein product is known to be expressed on B cells (Hogarth and Pietersz, 2012), in the CD32^+^CD4^+^ fraction, we hypothesized that this apparent lack of HIV DNA enrichment in the CD32^+^ fraction results from the disproportional presence of residual non-T cells in this fraction that might mask the effect. To prove this, we measured the expression of lineage marker mRNAs: *CD19* (a B cell marker) and *CD3D* and *CD3G* (T cell markers) in the CD32^+^CD4^+^ and CD32^−^CD4^+^ fractions. *CD19* mRNA was dramatically overrepresented in the CD32^+^ fraction (median enrichment, 37-fold, p < 0.0001) (Figure 4C). Remarkably, the *CD19* mRNA level in the CD32^+^ fraction negatively correlated with HIV DNA enrichment in this fraction (rho = −0.51, p = 0.029) (Figure 4D). Moreover, both *CD3D* and *CD3G* mRNAs were underrepresented in the CD32^+^ fraction (p < 0.0001) (Figures 4E and 4F). Although both *CD3* mRNAs were still more abundant than *CD19* mRNA in both fractions (Figures S3C and S3D), the T/B lineage marker ratio was much lower in the CD32^+^ fraction than the CD32^−^ fraction (8- versus 672-fold for median *CD3D*/*CD19* ratio and 6- versus 557-fold for median *CD3G*/*CD19* ratio, p < 0.0001 for both) (Figures S3E and S3F). The levels of *CD19* mRNA strongly correlated with the *CD32B* mRNA levels, confirming that CD32b^+^ cells were mostly B cells (rho = 0.86, p = 4.3 × 10^−6^) (Figure S3G).

Finally, the disproportional representation of non-CD4^+^ T cells in the CD32^+^ fraction compared with the CD32^−^ fraction was confirmed by flow cytometry (Figure S4A). Flow cytometry analysis performed following the isolation of the CD32^+^CD4^+^ T cells by magnetic sorting revealed a high proportion of non-CD4^+^T cells in the CD32^+^ fraction, whereas this proportion was low in the CD32^−^ fraction: note especially the large excess of B cells among the lymphocytes (middle panel). This is because although the amount of non-T cells remaining after one round of CD4^+^ T cell isolation is low, many of these non-T cells express CD32 and are therefore subsequently co-isolated with the CD32^+^CD4^+^ T cells.

These results suggested that enrichment for HIV DNA in the CD32^+^CD4^+^ fraction might be obscured by an excess of residual non-T cells in this fraction. To demonstrate this, we normalized HIV DNA to the T cell numbers, surrogated by the *CD3D* and *CD3G* mRNA levels. This resulted in modest but significant enrichment for HIV DNA in the CD32^+^ fraction (median enrichment, 2.7-fold; mean, 16-fold; p = 0.0005; and median, 2.5-fold; mean, 20-fold; p = 0.0003; when normalized to *CD3D* and *CD3G* mRNAs, respectively) (Figures 4G and 4H). In contrast, no enrichment for CA HIV US RNA was observed in the CD32^+^ fraction even after normalization to *CD3D* or *CD3G* mRNA (Figures S3H and S3I).

### An Additional Round of CD4^+^ T Cell Purification Leads to Significant HIV DNA Enrichment in the CD32^+^CD4^+^ Fraction

To further test our hypothesis that HIV DNA enrichment in the CD32^+^CD4^+^ T cell fraction is obscured by residual non-CD4^+^ T cells, we optimized our magnetic-sorting-based CD32^+^CD4^+^ T cell isolation protocol. By consecutively performing the CD4^+^ T cell negative selection twice, we were able to deplete most non-T cells present in the CD32^+^ fraction (Figure S4B). In particular, CD32^+^CD4^+^ cells were largely free from contaminating B cells (>97% CD19^−^) (Figure S4C). As shown in Figure S5, whereas the additional round of CD4^+^ T cell selection did not affect CD4^+^ T cell purity in the CD32^−^ fraction, total CD4^+^ T cell purity in the CD32^+^ fraction significantly improved after two consecutive rounds of CD4^+^ T cell selection compared with one round (p = 0.0071).

**Figure 5.**
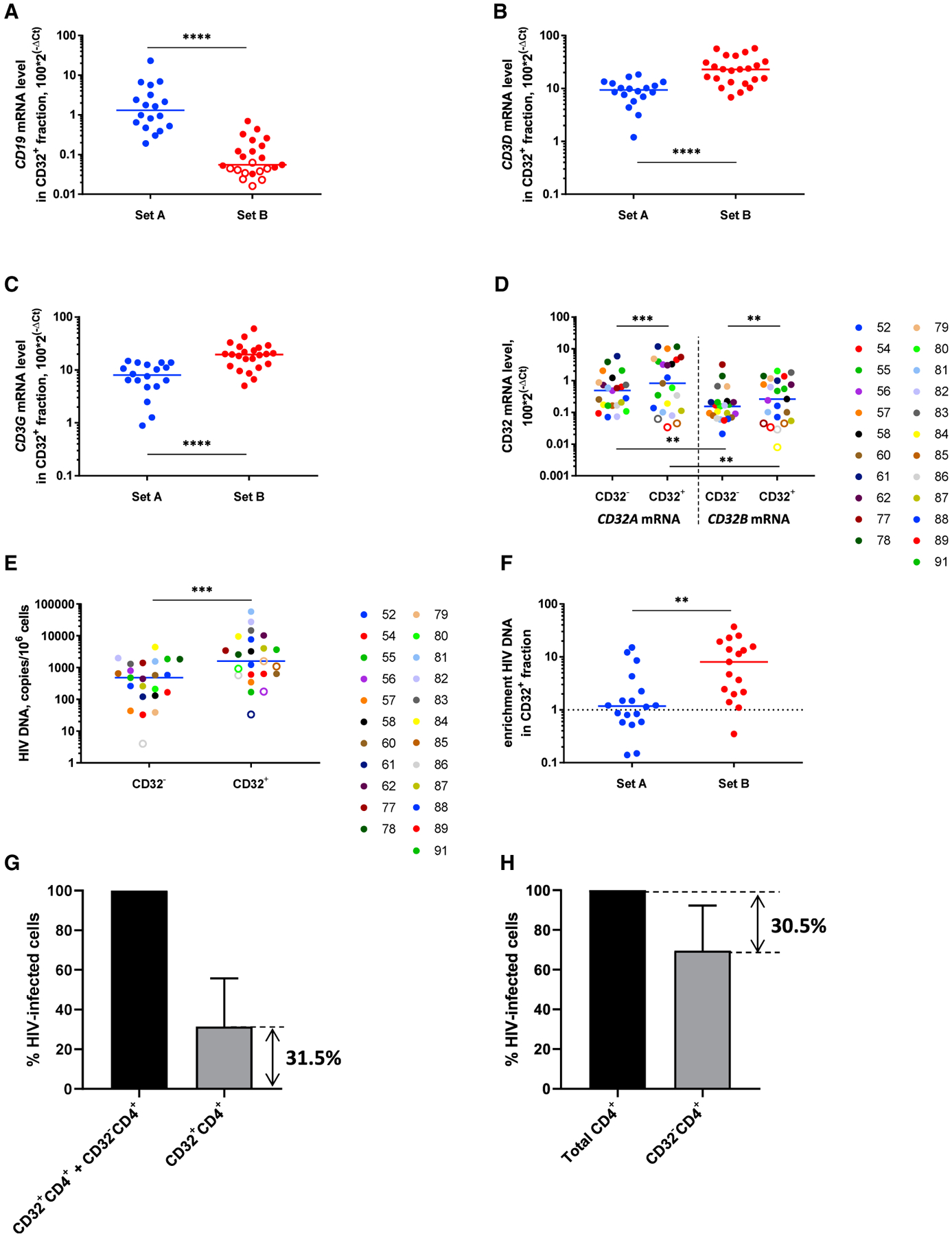
An Extra Round of CD4^+^ T Cell Purification Leads Both to a Reduction in Residual Non-T Cell Contribution to the CD32^+^ Fraction and to Significant Enrichment for HIV DNA in This Fraction (A–C) Comparison of *CD19* (A), *CD3D* (B), and *CD3G* (C) mRNA levels in the CD32^+^ fraction between set A (n = 18) and set B (n = 23). (D) *CD32A* and *CD32B* mRNA levels in CD32^+^ and CD32^−^ fractions of CD4+ T cells in set B. (E) HIV DNA levels, normalized to the total cellular DNA, in the CD32^+^ and CD32^−^ fractions, set B. (F) Comparison of HIV DNA enrichment in the CD32^+^ fraction between set A and set B. (G and H) Contribution of CD32^+^CD4^+^ cells to the total pool of HIV-infected cells, calculated based on a comparison of HIV DNA load either between CD32^+^CD4^+^ cells and CD32^−^CD4^+^ cells (n = 17) (G) or between total CD4^+^ cells and CD32^−^CD4^+^ cells after depletion of the CD32^+^ fraction (n = 7) (H). All mRNA levels were normalized to *GAPDH* mRNA. Open circles depict undetectable values, censored to the assay detection limits. The latter depended on the amounts of input cellular DNA or RNA and therefore differed between samples. Mann-Whitney tests (A–C and F) or Wilcoxon tests (D and E) were used to calculate statistical significance. ****p < 0.0001, ***0.0001 < p < 0.001, **0.001 < p < 0.01. Mean percentage values and SDs are shown (G and H). Only detectable values were included in the analyses shown in (F) and (G).

Using this optimized protocol, we isolated the CD32^+^ and CD32^−^CD4^+^ T cell fractions from 23 additional HIV-infected cART-treated individuals who had been aviremic for a median of 9 years (set B, Table S1). An additional round of CD4^+^ T cell purification resulted in a prominent decrease in the relative *CD19* mRNA levels in the CD32^+^ fraction, accompanied by an increase in the relative *CD3* mRNA levels, in set B compared with set A (p < 0.0001 for all mRNAs) (Figures 5A–5C). Accordingly, T/B lineage marker ratios dramatically increased in set B compared with set A (p < 0.0001) (Figures S6A–S6D). In set B, median *CD3*/*CD19* ratios in the CD32^+^ fraction became 199-fold for the *CD3D*/*CD19* ratio and 150-fold for the *CD3G*/*CD19* ratio. This indicated that the B cell contribution to the CD32^+^CD4^+^ fraction became negligible. Moreover, *CD19* mRNA was undetectable in 39% of the samples. In accordance with this, in set B, the relative *CD32B* mRNA levels in the CD32^+^ fraction were significantly lower than the *CD32A* mRNA levels (p = 0.0033) (Figure 5D; Figures S6E–S6H). *CD32A*/*CD32B* mRNA ratios in the CD32^+^ fraction were higher in set B than in set A (median, 2.0-fold versus 1.2-fold), but this difference did not achieve statistical significance (p = 0.097) and was more modest than the large difference in T/B lineage marker ratios between set A and set B.

Next, we determined HIV DNA levels in the CD32^+^CD4^+^ and CD32^−^CD4^+^ fractions in set B. In line with our hypothesis, we observed significant HIV DNA enrichment in the CD32^+^CD4^+^ fraction, even when HIV DNA was normalized to the total cellular DNA (median enrichment, 8.1-fold; mean, 10.9-fold; p = 0.0003) (Figure 5E; Figures S7A and S7B). This significant enrichment was also observed in a subset of set B participants with ≥4 years of suppressive cART (n = 17) (Figure S7C), reiterating the notion that CD32 is a marker of HIV latent reservoir. Therefore, optimization of the protocol to isolate a purer population of CD32^+^CD4^+^ cells resulted in significantly higher HIV DNA enrichment in these cells (p = 0.0018 for comparison of HIV DNA enrichment between set A and set B) (Figure 5F). We then used HIV DNA levels measured in the CD32^+^CD4^+^ and CD32^−^CD4^+^ fractions to calculate the contribution of CD32^+^CD4^+^ cells to the total HIV-infected cells, which averaged 31.5% (range, 2.3%–77.3%) (Figure 5G). To confirm this, we depleted CD32^+^ cells from the total CD4^+^ cells in a subset of set B participants (n = 7). The resulting reduction in HIV DNA level averaged 30.5% (Figure 5H), strikingly close to the percentage (31.5%) determined earlier. Significant enrichment for HIV DNA in the CD32^+^ fraction was also observed when the latter was normalized to *CD3D* or *CD3G* mRNA (median enrichment, 11.2-fold and 12.0-fold, respectively; p < 0.0001 for both normalizers) (Figures S7D and S7E).

### Additional Rounds of CD32 Positive Selection Lead to Further Progressive Enrichment for HIV DNA in the CD32^+^ Fraction

**Figure 6.**
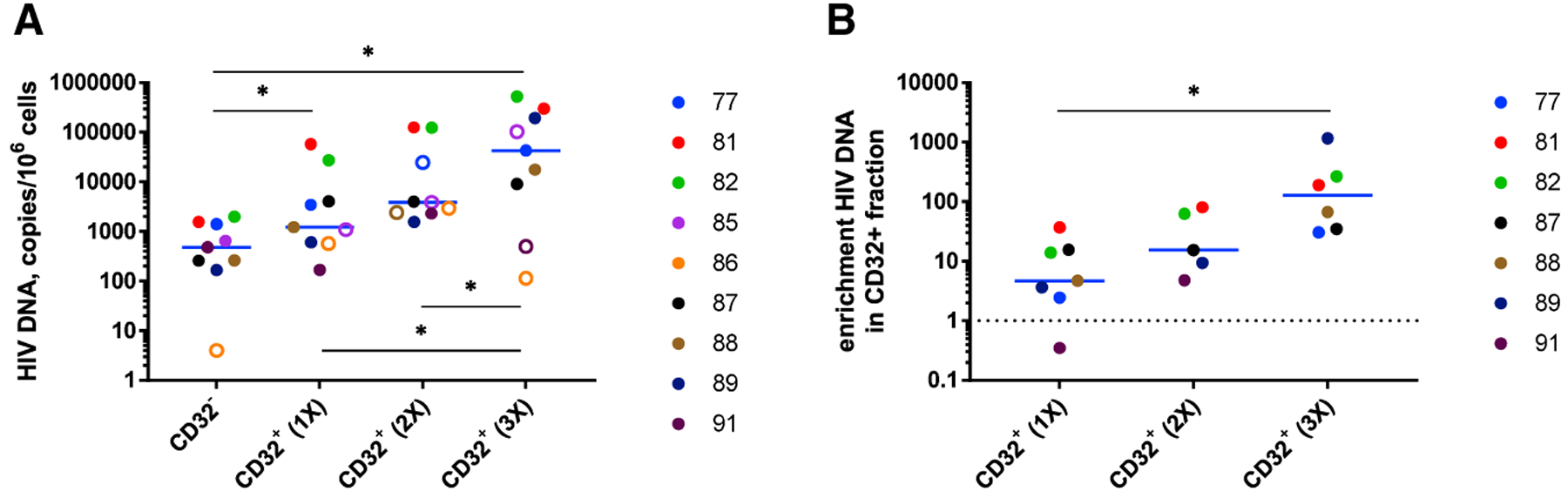
One or Two Extra Rounds of CD32 Positive Selection Lead to Further Progressive Enrichment for HIV DNA in the CD32^+^ Fraction (A) Comparison of HIV DNA levels, normalized to the total cellular DNA, between the CD32^−^ fraction and CD32^+^ fractions obtained after one, two, or three consecutive rounds of CD32 positive selection (n = 9). Open circles depict undetectable values, censored to the assay detection limits. The latter depended on the amounts of input cellular DNA and therefore differed between samples. (B) Comparison of HIV DNA enrichment in the CD32^+^ fractions obtained after one (n = 7), two (n = 5), or three (n = 6) consecutive rounds of CD32 positive selection. Only detectable values were included in the enrichment calculation. Wilcoxon tests were used to calculate statistical significance. *0.01 < p < 0.05.

As demonstrated earlier, enrichment for HIV DNA in CD32^+^CD4^+^ cells in set B was significant but still modest compared with enrichment reported by Descours et al. (2017). We hypothesized that this could be because one round of CD32 positive selection by magnetic sorting is insufficient to obtain a pure CD32^high^ cell population. Therefore, for nine participants of set B, we additionally performed one or two extra rounds of CD32 positive selection. This resulted in progressively increasing enrichment for HIV DNA in the CD32^+^ fraction: median enrichment was 4.7-fold (range, 0.4–37.0), 15.4-fold (4.8–80.3), and 129.5-fold (30.5–1,162.1) after one, two, or three consecutive rounds of CD32 positive selection (mean enrichment, 11.1-, 34.5-, and 292.4-fold, respectively) (Figure 6; Figure S7F). The latter value is in the same range as, albeit still somewhat lower than, the degree of enrichment reported by Descours et al. (2017) (median, 590-fold; mean, 1,025-fold). Remarkably, after three rounds of CD32 selection, the HIV DNA load reached 0.53 copies per cell (Figure 6), indicating a very high level of HIV infection of CD32^high^ cells. Similar degrees of enrichment were observed when HIV DNA was normalized to *CD3D* or *CD3G* mRNAs (Figures S8A–S8D).

### HIV Proviruses Are More Transcriptionally Silent in the CD32^+^ Fraction

**Figure 7.**
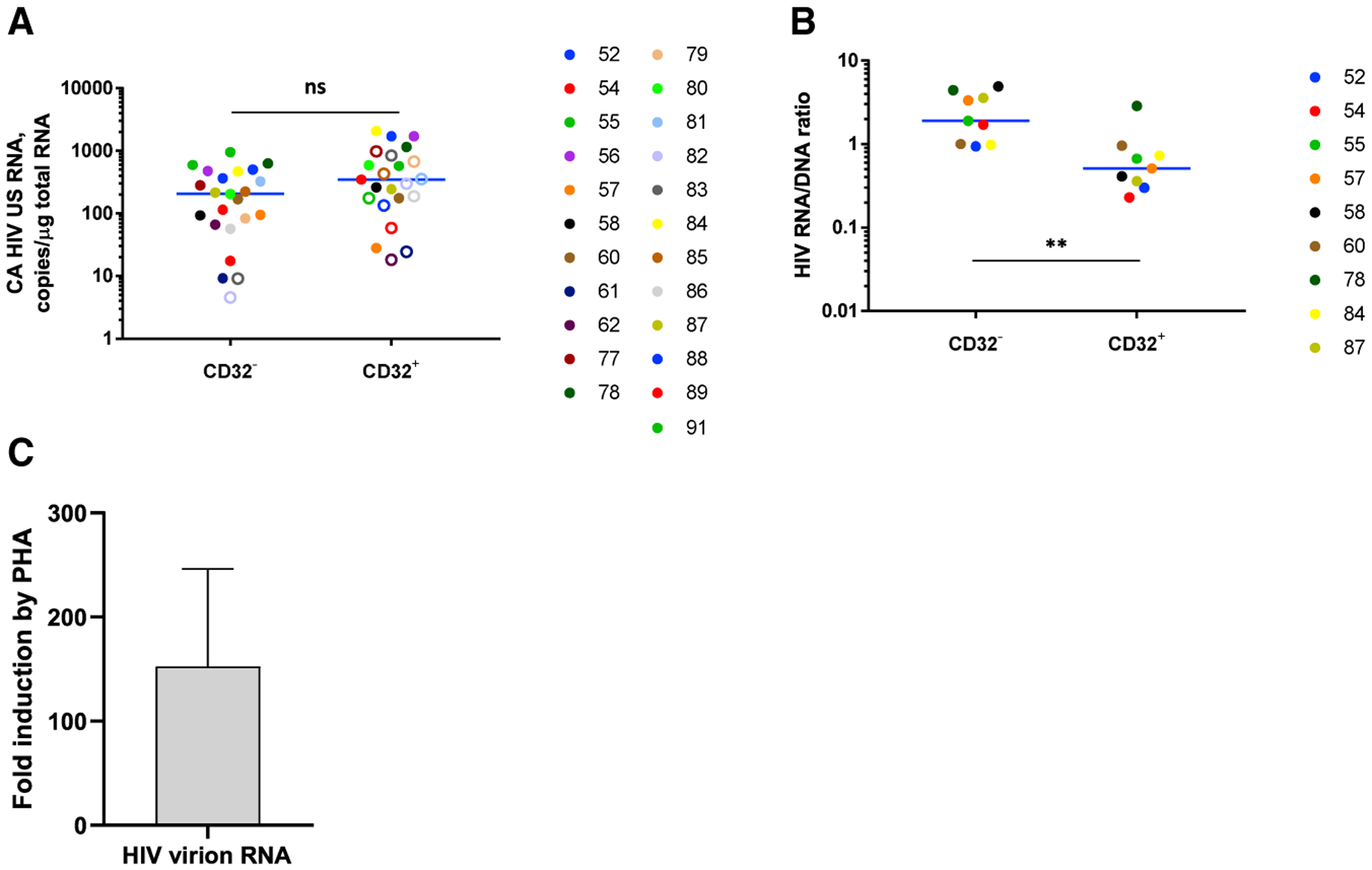
CD32^+^CD4^+^ Cells Harbor Latent Inducible HIV Proviruses (A) Comparison of CA HIV US RNA levels, normalized to the total cellular RNA, between CD32^−^ and CD32^+^ fractions (n = 23). Open circles depict undetectable values, censored to the assay detection limits. The latter depended on the amounts of input cellular RNA and therefore differed between samples. (B) Comparison of HIV RNA/DNA ratios between CD32^−^ and CD32^+^ fractions. Only samples in which both HIV DNA and RNA were detectable were included in the calculation (n = 9). Wilcoxon tests were used to calculate statistical significance. **0.001 < p < 0.01; ns, not significant. (C) Fold induction of extracellular HIV virion RNA production by PHA in *ex vivo* HIV reactivation assay (n = 5). Participant 93 with undetectable HIV RNA in both mock and PHA conditions was excluded from the calculation of fold induction (Figures S8G and S8H). Mean fold induction value and SEM are shown.

In contrast to HIV DNA, no enrichment for CA HIV US RNA in the CD32^+^CD4^+^ fraction was observed for set B (Figure 7A). Moreover, HIV RNA/DNA ratios, representing mean HIV transcriptional activity per provirus, were significantly lower in the CD32^+^ fraction (p = 0.0039) (Figure 7B). This indicated that HIV proviruses are significantly more transcriptionally silent in CD32^+^ cells compared with CD32^−^ cells. One could argue that this indicates transcriptional latency of the HIV proviruses, but it could also result from reduced RNA polymerase II activity in the CD32^+^ cells. We therefore measured the expression of glyceraldehyde 3-phosphate dehydrogenase (GAPDH), the product of a housekeeping gene, in the CD32^+^CD4^+^ and CD32^−^CD4^+^ fractions. Surprisingly, we did not observe reduced *GAPDH* mRNA expression in the CD32^+^ fraction (Figure S8E). Moreover, in the participants for whom we performed additional rounds of CD32 positive selection, we observed a significant progressive increase in *GAPDH* mRNA expression (Figure S8F), indicating that CD32^high^ cells are more transcriptionally active than CD32^−^ cells.

### CD32^+^CD4^+^ Cells Harbor Inducible HIV Proviruses

To assess whether CD32^+^CD4^+^ cells support HIV reactivation from latency, we performed an *ex vivo* HIV reactivation assay using CD32^+^CD4^+^ T cell fractions from six additional cART-treated, HIV-infected individuals who had been aviremic for a median of 6.5 years (participants 92, 93, and L16–L19, Table S1). *Ex vivo* cultures were mock treated or treated for 48 h with phytohemagglutinin (PHA) in the presence of dolutegravir to prevent viral spread. Reactivation of virus production was measured by quantitation of extracellular HIV virion RNA in culture supernatants. Compared with mock-treated cultures, PHA was able to reactivate virus production by a mean of 153-fold (Figures 7C; Figures S8G and S8H), demonstrating that despite low basal HIV transcription levels, CD32^+^CD4^+^ cells harbor inducible HIV proviruses that can be reactivated from latency to produce virus.

## DISCUSSION

The identification of a marker of latently infected cells has long been considered the holy grail of HIV cure programs. The discovery of such a marker would enable selective targeting and elimination of the HIV reservoir (Pillai and Deeks, 2017). A marker of latently infected cells could also facilitate dissection of the complex, heterogeneous, and dynamic nature of the reservoir and the molecular mechanisms of latency (Dahabieh et al., 2015). CD32a has been proposed as an HIV reservoir marker (Descours et al., 2017), but this finding has been challenged in subsequent reports. Abdel-Mohsen et al. (2018) observed that CD32 is mostly expressed in activated CD4^+^ T cells, and its expression is associated with a higher level of HIV transcription in the lymph nodes of HIV-infected individuals. However, in contrast to the Descours et al. (2017) study, Abdel-Mohsen et al. (2018) found no enrichment for HIV DNA in CD32^+^ cells from cART-suppressed HIV-infected individuals. Martin et al. (2018) and Badia et al. (2018) observed limited enrichment for HIV DNA in CD32^+^ cells in some participants but not in others and concluded that CD32 is not a specific marker of the HIV reservoir. Likewise, Pérez et al. (2018) and Osuna et al. (2018) could not demonstrate enrichment for HIV DNA in CD32^+^ cells.

The reason for this controversy may reside in the technical difficulty to obtain a sufficiently pure population of bona fide CD32^+^CD4^+^ T cells, as demonstrated by several studies. The frequency of CD32^+^ cells among APCs is much higher than among CD4^+^ T cells. Therefore, even if the residual APC contamination of CD4^+^ T cells is low in general, APCs will be disproportionally overrepresented in the CD32^+^ fraction. Furthermore, as shown by two independent groups, certain cell-sorting strategies and/or settings might result in the isolation of T-B cell doublets or conjugates instead of bona fide CD32^+^CD4^+^ T cells (Osuna et al., 2018; Thornhill et al., 2019). Both cell subsets are rare, but T-B cell conjugates highly express CD32b on the B cell part and CD4 on the T cell part; therefore, these cells might preferentially be isolated by simultaneous gating for CD4 and CD32. In contrast, bona fide CD32^+^CD4^+^ cells, which likely express less CD32 per cell, might be underrepresented or missed, unless non-T cells are thoroughly depleted prior to CD32 staining. For example, although Badia et al. (2018) used CD14 staining to exclude monocytes from isolated CD4^+^ T cells of HIV-infected individuals, they did not specifically deplete cells expressing a B cell marker; therefore, T-B cell doublets and conjugates were not excluded. No specific post-sort purities of CD4^+^ cells from the B cell contamination were reported in their study. It should be noted that Pérez et al. (2018) made an effort to exclude the non-T cell contaminants (including B cells) by additional gating; however, post-sort flow cytometry revealed that on average, only 22.5% of the CD32^high^ cell population and 2.5% of the CD32^int^ cell population were CD32^+^CD4^+^ cells. Moreover, specific post-sort purities of CD4^+^ cells from the B cell contamination were not reported in their study (Pérez et al., 2018). Therefore, it cannot be excluded that even those 22.5% of CD32^high^ cells that were CD32^+^CD4^+^ were partly or mostly composed of T-B cell doublets and conjugates.

Our magnetic cell-sorting strategy allowed us to avoid these problems, because two rounds of CD4^+^ T cell negative selection could efficiently deplete most contaminating non-T cells, including possible T-non-T cell conjugates. This allowed us to obtain a purified population of CD4^+^ cells before we started with CD32^+^ cell isolation. The sequential sorting strategy was likely responsible for the apparent absence of T-B cell conjugates from our CD32^+^CD4^+^ cell population. In contrast to the study of Osuna et al. (2018), in which a ratio of 1:1 between *CD3G* and *CD19* mRNAs (T and B cell markers, respectively) was observed in the CD32^+^ fraction, in our study, this median ratio was 6:1 after one round and at least 150:1 after two rounds of CD4^+^ negative selection. Moreover, HIV DNA enrichment in the CD32^+^ fraction negatively correlated with the B cell-specific *CD19* mRNA levels in this fraction, and the second CD4^+^ negative selection round caused both at least a 22-fold drop in *CD19* mRNA levels and a 7-fold increase in HIV DNA enrichment in the CD32^+^ fraction. We also directly demonstrated that our CD32^+^CD4^+^ cells were largely free from B cell contamination (<3% of CD32^+^CD4^+^ cells were CD19^+^). In agreement with this, although *CD32A* mRNA could not be detected in CD32^+^ cells in the Osuna et al. (2018) study, it was readily detectable in our study, being at a median of 1.2-fold and 2.0-fold more abundant than *CD32B* mRNA after one and two rounds of CD4^+^ selection, respectively. However, even two rounds of CD4^+^ selection did not remove the *CD32B* mRNA completely, indicating that some CD4^+^ T cells might express CD32b. This is in line with the findings of Holgado et al. (2018), who demonstrated expression of both *CD32A* and *CD32B* mRNAs in CD4^+^ T cells, with an excess of *CD32A*. Therefore, although Descours et al. (2017) reported the selective induction of CD32a but not CD32b upon HIV infection, we were unable to discriminate between the two CD32 isoforms for the association with HIV DNA enrichment.

Although CD4^+^ negative selection could efficiently deplete the contaminating non-T cells, our study demonstrated that one round of CD32 positive selection was insufficient to obtain a pure CD32^high^ cell population. Only when we repeated the CD32 positive selection two extra times did we observe high enrichment for HIV DNA in the CD32^+^ fraction. Although the degree of enrichment was still ~4 times lower than that observed by Descours et al. (2017), we are confident that we have confirmed their findings using a different method, which adds value to these results. In addition to their main groundbreaking finding of high enrichment for HIV DNA in CD32^+^ cells, Descours et al. (2017) also demonstrated enrichment for replication-competent proviruses in these cells. However, the degree of enrichment for replication-competent proviruses in their study, albeit high in absolute terms, was not higher than that of enrichment for total HIV DNA, suggesting that CD32^+^ cells are not specifically enriched for replication-competent proviruses. Although we confirmed high enrichment for HIV DNA in CD32^+^ cells and demonstrated that these proviruses can be reactivated *ex vivo* to produce virus particles, it is technically challenging to exactly determine the extent to which these proviruses are replication competent. The only definitive assay allowing for unequivocal establishment of the replication competence of a provirus is quantitative viral outgrowth assay (qVOA). However, although qVOA has long been considered the gold standard for measurement of replication-competent HIV, it requires large cell numbers and has been shown to underestimate the amount of replication-competent proviruses by 1–2 orders of magnitude (Bruner et al., 2016; Ho et al., 2013). Therefore, one could expect false-negative wells, especially when starting from very low cell numbers, as expected for the CD32^+^ fraction. This might have been one reason other groups could not confirm the results of Descours et al. (2017) (Badia et al., 2018; Bertagnolli et al., 2018). Alternative approaches, possibly including full-length, single-genome sequencing of HIV proviruses in CD32^+^ cells or the recently developed intact proviral DNA assay (IPDA) (Bruner et al., 2019; Hiener et al., 2017), might be used in the future to fully characterize HIV persistence in these cells. However, these techniques are also challenging to perform on low HIV DNA inputs, and one should realize that the lack of obvious genetic defects as determined by provirus sequencing or other methods still does not guarantee replication competence because not all defects can be determined by sequence analysis.

Apart from confirming high enrichment for HIV DNA in CD32^+^ cells, our report presents three findings: (1) frequencies of CD32^+^CD4^+^ cells positively correlate with the HIV DNA load in PBMCs, (2) HIV proviruses in CD32^+^ cells are more transcriptionally silent than in CD32^−^ cells, and (3) despite these low basal HIV transcription levels, CD32^+^CD4^+^ cells harbor latent, inducible HIV proviruses that can be reactivated from latency to produce virus. The low basal HIV transcription levels in CD32^+^CD4^+^ cells may seem somewhat paradoxical because we and others (Abdel-Mohsen et al., 2018; Martin et al., 2018; Noto et al., 2018; Wittner et al., 2018) have demonstrated that most CD32^+^CD4^+^ T cells express the activation marker HLA-DR. Moreover, we found that CD32^high^ cells are more transcriptionally active than CD32^−^ cells, which is in accordance with their activated phenotype. This suggests the presence of a specific mechanism of transcriptional silencing of HIV proviruses in these cells. Although further studies are needed to elucidate whether CD32 signaling is involved in HIV transcriptional silencing, contribution of activated cells to HIV persistence has been reported previously (Chavez et al., 2015; Chun et al., 2005; Deeks et al., 2016; Hatano et al., 2013; Kaiser et al., 2007; Khoury et al., 2016; Lee et al., 2019; Murray et al., 2014). Chavez et al. (2015) have demonstrated that HIV latency can be established in activated CD4^+^ T cells without returning to a resting state. Murray et al. (2014) indicated that some activation occurs within the HIV reservoir in cART-treated individuals. The latter study found total, episomal, and integrated HIV DNA in activated memory CD4^+^ T cells long after the decrease in cellular activation that accompanies suppression of viral replication with cART. Chun et al. (2005) have shown that total HIV DNA was present in activated CD4^+^ T cells at a level higher than in resting cells, despite individuals receiving cART for a mean of 9.1 years. Likewise, Kaiser et al. (2007) demonstrated higher HIV DNA levels in the activated compared with resting CD4^+^ T cells in cART-treated patients. Our group also demonstrated that HIV can establish latent infection in actively proliferating primary T lymphocytes and that co-culturing with dendritic cells reversed HIV latency (van der Sluis et al., 2013, 2015; van Montfort et al., 2019). The existence of latently infected CD4^+^ cells that are activated, and therefore relatively short-lived, suggests continuous replenishment of this component of the reservoir by cellular proliferation (Chun et al., 2005). Therefore, future studies should determine the clonality of HIV proviruses in CD32^+^ cells, as well as the integration status of HIV DNA in these cells.

Recently, several studies have demonstrated an association of CD32 expression with HIV transcriptional activity in the lymph node and gut tissue (Abdel-Mohsen et al., 2018; Noto et al., 2018; Vásquez et al., 2019), contrasting with our observation that most HIV proviruses in peripheral blood CD32^+^ cells are transcriptionally silent. Although these discrepant results may reflect the specific assays used (*in situ* hybridization-based versus qPCR-based CA HIV RNA measurements), they might also suggest differences in mechanisms of persistence of HIV-infected CD32^+^ cells between peripheral blood and tissues. Residual HIV replication in tissue compartments can be prominent because of low antiretroviral drug penetration (Estes et al., 2017; Fletcher et al., 2014; Lorenzo-Redondo et al., 2016; Rothenberger et al., 2019), and CD32 expression in CD4^+^ T cells is induced upon *in vitro* HIV infection (Abdel-Mohsen et al., 2018; Badia et al., 2018; Descours et al., 2017; Grau-Expósito et al., 2017). Therefore, in tissues, CD32 might mark cells that were recently infected despite cART. Alternatively, CD32 expression might be upregulated upon reactivation of HIV transcription in tissues. Mechanisms of CD32 expression in peripheral blood cells harboring transcriptionally silent HIV proviruses remain to be investigated but can reflect a role of CD32 in maintaining persistence of HIV reservoir by clonal proliferation.

In addition to true viral latency, lower CA HIV RNA levels in infected cells can be caused by the presence of proviruses with genetic defects in the HIV promoter region or the Tat protein, which would preclude high-level CA RNA transcription. However, it is unlikely that defective proviruses are the major cause of lower HIV transcription levels in CD32^+^ cells for several reasons. First, it has been shown that the relative amounts of defective proviruses are not higher in CD32^+^ cells compared with CD32^−^ cells (Descours et al., 2017). Second, several studies have shown that defective proviruses can transcribe HIV RNA (Imamichi et al., 2016; Pollack et al., 2017). Moreover, in cART-treated individuals, they transcribe more HIV RNA than intact proviruses (Barton et al., 2016; Wiegand et al., 2017; Winckelmann et al., 2017), which is in line with the selective elimination of cells harboring CA RNA-expressing intact proviruses by the host immunity (Pinzone et al., 2019). Third, we demonstrated here that HIV proviruses in CD32^+^CD4^+^ cells can be induced *ex vivo* to produce virus, strongly suggesting that these cells harbor replication-competent proviruses. Altogether, this suggests that the preponderance of latent, not defective, proviruses in CD32^+^ cells is the major reason behind lower HIV transcription levels in these cells.

As discussed earlier, insufficient purity of the isolated CD32^+^CD4^+^ fraction and/or isolation of T-B cell conjugates instead of bona fide CD32^+^CD4^+^ cells are likely some of the reasons other groups did not observe the HIV DNA enrichment in CD32^+^ cells. Another possible reason could be underdetection of HIV DNA in the CD32^+^ fraction because of DNA losses during extractions from low cell numbers. Indeed, HIV DNA was undetectable in up to 60%–80% of CD32^high^ samples in some other studies (Osuna et al., 2018; Pérez et al., 2018). In our study, we solved this problem by adding carrier RNA to every extraction, which allowed us to extract DNA from very low cell numbers without losses and to prevent possible artificial overquantitation of HIV DNA relative to cellular DNA when working with low cellular inputs into qPCR. This resulted in HIV DNA detectability of 100% and 74% in the set A and set B samples, respectively, even though we started from much lower PBMC numbers than other groups, who used leukapheresis samples (Osuna et al., 2018; Pérez et al., 2018). At the same time, we prevented artificial underquantitation of HIV DNA by avoiding the use of high cellular DNA amounts in qPCR. These technical amendments allowed the accurate quantitation of HIV DNA in both CD32^+^ and CD32^−^ fractions.

In summary, we confirmed that CD32^+^CD4^+^ T cells are highly enriched for HIV proviruses and directly demonstrated that HIV DNA enrichment in these cells depends on the purity of the CD32^+^CD4^+^ T cell fraction. This provides a plausible explanation for the failure of others to detect HIV DNA enrichment in these cells and underscores the importance of isolating pure, bona fide CD32^+^CD4^+^ T cells for future studies. We further demonstrated that CD32^+^CD4^+^ T cells often co-express ICs, suggesting an overlap between the CD32^+^CD4^+^ T cell reservoir and the reservoir identified by ICs (Fromentin et al., 2016). Our findings replenish the hope that CD32, besides other markers such as CXCR3 (Banga et al., 2018), could facilitate the quest for a cure for HIV infection.

## STAR⋆METHODS

### LEAD CONTACT AND MATERIALS AVAILABILITY

Further information and requests for resources and reagents should be directed to and will be fulfilled by the Lead Contact, Alexander O. Pasternak (a.o.pasternak@amsterdamumc.nl). This study did not generate new unique reagents.

### EXPERIMENTAL MODEL AND SUBJECT DETAILS

Fifty-five HIV-infected aviremic cART-treated individuals were included in the study: 51 were monitored in the Amsterdam University Medical Center (#10–95, Table S1) and four in the Liège University Hospital (#L1-L4, Table S1). For Amsterdam participants, PBMCs were isolated from the buffy coats that were left after removal of plasma from blood samples (24 mL), which were originally taken for routine diagnostic/follow-up purposes (plasma viral load measurements). All samples were processed anonymously and the study has been conducted in accordance with ethical regulations summarized in the Code for Proper Secondary Use of Human Tissue in the Netherlands (https://www.federa.org/codes-conduct), developed by the Federation of Dutch Medical Scientific Societies (FMWV). For Liège participants, who donated 120 mL of blood, the study has been approved by the Ethical Committee of the Liège University Hospital and all participants provided informed consent. Characteristics of participants are presented in Table S1.

### METHOD DETAILS

#### PBMC isolation and flow cytometry

PBMCs were isolated from blood of HIV-infected individuals by use of a Ficoll gradient. PBMC isolation and magnetic sorting were performed in all 55 individuals, CD32 FACS staining and appropriate FMO control in 17 individuals (Set A), and complete phenotype analysis (ICs, CD2, as well as respective FMO controls) in 7 individuals (Set A), in order to keep sufficient cell numbers for magnetic sorting. In Set A, 13 samples were processed from PBMCs cryopreserved for various periods (days to months), and 5 were processed from fresh blood. No significant difference was observed between frozen and fresh PBMC samples in percentages of CD32^+^ cells (determined by either flow cytometry or magnetic sorting) or in enrichment for HIV DNA in CD32^+^ fraction (data not shown). In Set B, all 23 samples were directly processed from fresh blood. PBMCs were washed and stained with monoclonal antibodies (mAbs) for 30 minutes at 4°C in the dark, to determine expression of different surface molecules. The following antibody panels were used to measure the expression of CD32, HLA-DR, CD2, CD19, and ICs in total CD4^+^ T cells or CD32^+^CD4^+^ cells: CD3-BV711 (Biolegend), CD4-BUV395 (BD Biosciences), CD8-BUV496 (BD Biosciences), HLA-DR-PE-Cy7 (BD Biosciences), CD2-BB700 (BD Biosciences), CD32-APC clone FUN-2 (SONY Biotechnology), PD-1-APC/Fire750 (Biolegend), TIGIT-PE (Biolegend), LAG-3-BV421 (Biolegend), CD19-Pacific Blue (Biolegend). For expression of CD32 and all ICs, gates were defined using FMO controls. For CD2, CD19, and HLA-DR, the same threshold was used for all participants. Fluorescence was measured with the BD LSR Fortessa cell analyzer. Analyses were performed with the analysis platform FlowJo V10.

#### Magnetic cell sorting

Total CD4^+^ T cells were isolated from PBMCs of HIV-infected individuals by negative selection using the CD4^+^ T Cell Isolation Kit (MACS, Miltenyi Biotec) on MACS LS columns. This kit contains the cocktail of monoclonal antibodies against CD8, CD14, CD15, CD16, CD19, CD36, CD56, CD123, TcRγ/δ, and CD235a. Total CD4^+^ T cells were then stained with APC anti-human CD32 antibody (clone FUN-2, SONY). CD32^+^CD4^+^ T cells were subsequently magnetically labeled with anti-APC microbeads (MACS, Miltenyi Biotec) and isolated by positive selection on MACS MS columns. Sorted CD32^+^CD4^+^ and CD32^−^CD4^+^ T cell fractions were either directly used for HIV DNA and RNA measurements or for the HIV reactivation assay (see below). The relative sizes of the sorted CD32^+^CD4^+^ and CD32^−^CD4^+^ T cell fractions were determined by cellular DNA quantitation in these fractions by qPCR using β-actin detection reagents (Thermo Fisher Scientific) and expressed as percentages of total CD4^+^ T cells.

#### *Ex vivo* HIV reactivation assay

CD32^+^CD4^+^ cells isolated by magnetic sorting as described above were either cultured in RPMI alone or stimulated with 10 mg/mL PHA for 48 hours in the presence of dolutegravir (100 nM). After 48 hours, culture supernatants were harvested and lysed in 900 mL of L6 lysis buffer (Boom et al., 1990) for subsequent isolation and quantitation of extracellular HIV virion RNA.

#### Nucleic acid extraction and quantitation

Nucleic acids were extracted from total PBMCs, sorted CD4^+^ T cell fractions, or culture supernatants using the Boom isolation method (Boom et al., 1990) with the addition of two micrograms of Poly-A Carrier RNA (QIAGEN) to the lysed cells or extracellular lysates prior to the silica binding step. For cell-derived material, extracted RNA was treated with DNase (DNA-free kit; Thermo Fisher Scientific) to remove DNA that could interfere with the quantitation. Cellular RNA was reverse transcribed using random primers and SuperScript III reverse transcriptase (all from Thermo Fisher Scientific), and HIV DNA and RNA were quantified by qPCR using a previously described assay (Malnati et al., 2008). HIV DNA or RNA copy numbers were determined using a 7-point standard curve with a linear range of more than 5 orders of magnitude that was included in every qPCR run (R^2^ > 0.996 for all measurements), and normalized to the total cellular DNA (by measurement of β-actin DNA) or RNA (by measurement of 18S ribosomal RNA) inputs, respectively, as described previously (Pasternak et al., 2009). Alternatively, HIV DNA was normalized to T cell numbers, surrogated by *CD3D* and *CD3G* mRNA levels.

To quantify extracellular HIV virion RNA in the HIV reactivation assay, the total volume of extracted extracellular RNA was subjected to one-step reverse transcription (RT)-PCR using primers that amplify a region within the HIV *gag* gene (primers GAG1 and SK431; Pasternak et al., 2008). One-step RT-PCR was performed using QIAGEN OneStep RT-PCR Kit according to manufacturer’s instructions. An aliquot from this RT-PCR reaction was subsequently used as template for seminested qPCR as previously described (Pasternak et al., 2008).

Cellular mRNAs were quantified using TaqMan Gene Expression Assays (all from Thermo Fisher Scientific): *CD32A*: Hs01013401_g1, *CD32B*: Hs00269610_m1, *CD3D*: Hs00174158_m1, *CD3G*: Hs00173941_m1, *CD19*: Hs00174333_m1, *GAPDH*: Hs02758991_g1.

For all assays, non-template control wells were included in every qPCR run and were consistently negative.

### QUANTITATION AND STATISTICAL ANALYSIS

Data were analyzed using Prism 8.0.2 (GraphPad Software). Non-parametric analyses (unpaired Mann-Whitney tests, paired Wilcoxon tests, Friedman tests with Dunn’s post-tests, or Spearman correlations) were used throughout the manuscript as appropriate (individual tests are described in Figure Legends). Undetectable values were left-censored to the assay detection limits. The latter depend on the amounts of the normalizer (input cellular DNA or RNA), and therefore differed between samples. Undetectable values were included in unpaired Mann-Whitney tests without restrictions. For paired Wilcoxon tests, pairs were included in the analysis only when either (i) both values in a pair were detectable, or (ii) one value in a pair was undetectable and the other detectable, and the maximal value of the undetectable (the assay detection limit) was lower than the detectable. Same rules were applied for quantitation of the fold induction of extracellular HIV virion RNA by PHA in the *ex vivo* HIV reactivation assay. As a sensitivity analysis, we also performed Wilcoxon tests including only pairs where both values were detectable. All statistical tests were two-sided and p values < 0.05 were considered statistically significant.

### DATA AND CODE AVAILABILITY

All data are available from the corresponding authors upon reasonable request.

## Supplementary Material

1

2

## Figures and Tables

**Table:** KEY RESOURCES TABLE

REAGENT or RESOURCE	SOURCE	IDENTIFIER
Antibodies		
Brilliant Violet 711 anti-human CD3, clone OKT3	BioLegend	Cat#317328; RRID:AB_2562907
BUV395 Mouse Anti-Human CD4, Clone RPA-T4	BD Biosciences	Cat#564724; RRID:AB_2738917
BUV496 Mouse Anti-Human CD8, Clone RPA-T8	BD Biosciences	Cat#612942; RRID:AB_2744460
PE-Cy7 Mouse Anti-Human HLA-DR, Clone G46–6	BD Biosciences	Cat#560651; RRID:AB_1727528
BB700 Mouse Anti-Human CD2, Clone S5.2	BD Biosciences	Cat#746066; RRID:AB_2743447
APC/Fire 750 anti-human CD279 (PD-1), clone EH12.2H7	BioLegend	Cat#329954; RRID:AB_2616721
PE anti-human TIGIT (VSTM3), clone A15153G	BioLegend	Cat#372704; RRID:AB_2632730
Brilliant Violet 421 anti-human CD223 (LAG-3), clone 11C3C65	BioLegend	Cat#369314; RRID:AB_2629797
APC anti-human CD32, clone FUN-2	Sony Biotechnology	Cat#2116040
Pacific Blue anti-human CD19, clone HIB19	BioLegend	Cat#302232; RRID:AB_2073118
Biological Samples		
PBMC samples from HIV-infected individuals	Amsterdam UMC, Liège University Hospital	N/A
Chemicals, Peptides, and Recombinant Proteins		
Phytohemagglutinin-M (PHA-M)	Sigma-Aldrich	Cat#11082132001
Poly-A Carrier RNA	QIAGEN	Cat#1048147
SuperScript III reverse transcriptase	ThermoFisher Scientific	Cat#18080–085
Random Primers	ThermoFisher Scientific	Cat#48190–011
RNaseOUT Recombinant Ribonuclease Inhibitor	ThermoFisher Scientific	Cat#10777–019
Dolutegravir	ViiV Healthcare	N/A
Critical Commercial Assays		
CD4^+^ T Cell Isolation Kit, human	Miltenyi Biotec	Cat#130-096-533
Anti-APC MicroBeads	Miltenyi Biotec	Cat#130-090-855
TaqMan β-Actin Detection Reagents	ThermoFisher Scientific	Cat#401846
QIAGEN OneStep RT-PCR Kit	QIAGEN	Cat#210212
DNA-*free* DNA Removal Kit	ThermoFisher Scientific	Cat#AM1906
Platinum Quantitative PCR SuperMix-UDG	ThermoFisher Scientific	Cat#11730–025
TaqMan Ribosomal RNA Control Reagents	ThermoFisher Scientific	Cat#4308329
TaqMan Gene Expression Assay, CD32A (Hs01013401_g1)	ThermoFisher Scientific	Cat#4331182
TaqMan Gene Expression Assay, CD32B (Hs00269610_m1)	ThermoFisher Scientific	Cat#4331182
TaqMan Gene Expression Assay, CD3D (Hs00174158_m1)	ThermoFisher Scientific	Cat#4331182
TaqMan Gene Expression Assay, CD3G (Hs00173941_m1)	ThermoFisher Scientific	Cat#4331182
TaqMan Gene Expression Assay, CD19 (Hs00174333_m1)	ThermoFisher Scientific	Cat#4331182
TaqMan Gene Expression Assay, GAPDH (Hs02758991_g1)	ThermoFisher Scientific	Cat#4331182
Oligonucleotides		
Malnati_inner_F (TCTCGACGCAGGACTCG)	Malnati et al., 2008	https://www.nature.com/articles/nprot.2008.108
Malnati_inner_R (TACTGACGCTCTCGCACC)	Malnati et al., 2008	https://www.nature.com/articles/nprot.2008.108
Malnati_probe (6FAM-CTCTCTCCTTCTAGCCTC-MGBNFQ)	Malnati et al., 2008	https://www.nature.com/articles/nprot.2008.108
3AG1 (TCAGCCCAGAAGTAATACCCATGT)	Pasternak et al., 2008	https://jcm.asm.org/content/46/7/2206.long
SK431 (TGCTATGTCAGTTCCCCTTGGTTCTCT)	Pasternak et al., 2008	https://jcm.asm.org/content/46/7/2206.long
3AG2 (CACTGTGTTTAGCATGGTGTTT)	Pasternak et al., 2008	https://jcm.asm.org/content/46/7/2206.long
3AG3 (FAM-ATTATCAGAAGGAGCCACCCCACAAGA-TAMRA)	Pasternak et al., 2008	https://jcm.asm.org/content/46/7/2206.long
Recombinant DNA		
pLAIΔRT	Pasternak et al., 2008	https://jcm.asm.org/content/46/7/2206.long
pGAG2-A5	Pasternak et al., 2008	https://jcm.asm.org/content/46/7/2206.long
Software and Algorithms		
GraphPad Prism 8.0.2	GraphPad Software	https://www.graphpad.com/
7000 System SDS Software v1.2.3	Applied Biosystems	https://resource.thermofisher.com/pages2013/WE111944
FlowJo v10	Becton, Dickinson & Company	https://www.flowjo.com/
Other		
LS Columns	Miltenyi Biotec	Cat#130-042-401
MS Columns	Miltenyi Biotec	Cat#130-042-201

## Highlights

- Percentages of CD32^+^ cells among CD4^+^ T cells correlate with HIV DNA loads in PBMC
- High enrichment for HIV DNA in purified CD32^+^CD4^+^ cells from cART-treated patients
- HIV transcription level per provirus is reduced in patient-derived CD32^+^CD4^+^ cells
- HIV proviruses in purified ^CD32^+^CD4^+ cells can be reactivated *ex vivo* to produce virus
