# Supplementary material for: CD32+CD4+ T Cells Are Highly Enriched for HIV DNA and Can Support Transcriptional Latency
Source: Cell Rep. Author manuscript; Available in PMC 2020 Mar 2. (PMC7050565; doi:10.1016/j.celrep.2020.01.071)
Supplement: 1 [file NIHMS1563621-supplement-1.pdf]

Cell Reports, Volume *30*

Supplemental Information

CD32^{+}CD4^{+} T Cells Are Highly Enriched for HIV DNA and Can Support Transcriptional Latency

Gilles Darcis, Neeltje A. Kootstra, Berend Hooibrink, Thijs van Montfort, Irma Maurer, Kevin Groen, Suzanne Jurriaans, Margreet Bakker, Carine van Lint, Ben Berkhout, and Alexander O. Pasternak

Table S1. Study participants. Related to Figures 1-7.

	Code	Gender	Age (years)	Current treatment	Duration of undetectable viral load (years)
Set A (Figures 1-4)	10	M	35	TDF/FTC/RLP	3
	11	F	45	ABC/3TC/DTG	5
	13	F	55	DRV/r DTG	15
	15	M	45	TDF/FTC/NVP	6
	16	M	43	TDF/FTC/EVG/c	4
	17	F	36	ABC/3TC/DTG	15
	18	F	46	TDF/FTC/EFV	15
	19	M	54	TDF/DRV/r/MVC	8
	20	M	59	TDF/FTC/EFV	5
	21	F	47	TDF/FTC/EFV	10
	22	F	46	TDF/FTC/RAL	18
	25	M	64	TDF/FTC/EVG/c	1
	26	M	52	ABC/3TC/DTG	10
	27	F	40	TDF/FTC/DRV/r	0.3
	28	M	46	TDF/FTC/EFV	2
	34	M	43	TDF/FTC/NVP	9
	36	F	37	TDF/FTC/DRV/r	2
	37	M	43	TDF/FTC/DRV/r	13
Set B (Figures 5-7)	52	M	67	TDF/FTC/ATZ/r	13
	54	M	45	ABC/3TC/DRN/r	2
	55	F	48	3TC/ZDV/LPV/r	2
	56	F	72	TDF/FTC/ETV/RAL	11
	57	M	60	ABC/3TC/RAL	8
	58	F	37	TDF/FTC/NVP	10
	60	M	63	RAL/MVC/DRV/c	14
	61	M	43	ABC/3TC/DTG	0.4
	62	F	46	ABC/3TC/DTG	2
	77	M	66	ABC/3TC/DTG	20
	78	M	55	ABC/3TC/DTG	4
	79	F	41	TDF/FTC/ATZ/r	4
	80	F	37	TAF/FTC/EVG/c	8
	81	M	46	TDF/FTC/NVP	3
	82	M	33	ABC/3TC/DTG	3
	83	M	56	TDF/FTC/RAL	21
	84	M	66	TDF/FTC/EFV	18
	85	M	64	TDF/FTC/EFV	10
	86	M	39	ABC/3TC/DTG	9
	87	F	56	ABC/3TC/EFV	11
	88	F	45	ABC/3TC/DRV/r	11
	89	M	43	TDF/FTC/DRV/r	4
	91	M	61	DTG/DRV/c	20
Additional participants (Figures 7C, S4, S5, S8H)	31	M	72	TDF/FTC/EVG/c	1.9
	32	M	46	TDF/FTC/RIL	8.2
	33	M	30	ABC/3TC/DTG	<0.4
	45	M	55	TDF/FTC/EVG/c	0.4
	46	M	47	TDF/FTC/RIL	4.4
	47	F	38	ABC/3TC/DRV/r	0.4
	92	M	53	TDF/FTC/EVG/c	6
	93	M	49	TDF/FTC/EVG/c	7
	94	M	18	ABC/3TC/DTG	4
	95	F	44	TAF/FTC/BIC	<0.4
	L16	M	71	TAF/FTC/RPV	3
	L17	F	45	TAF/FTC/RPV	7
	L18	M	37	ABC/3TC/DTG	4
	L19	M	52	TAF/FTC/NVP	14

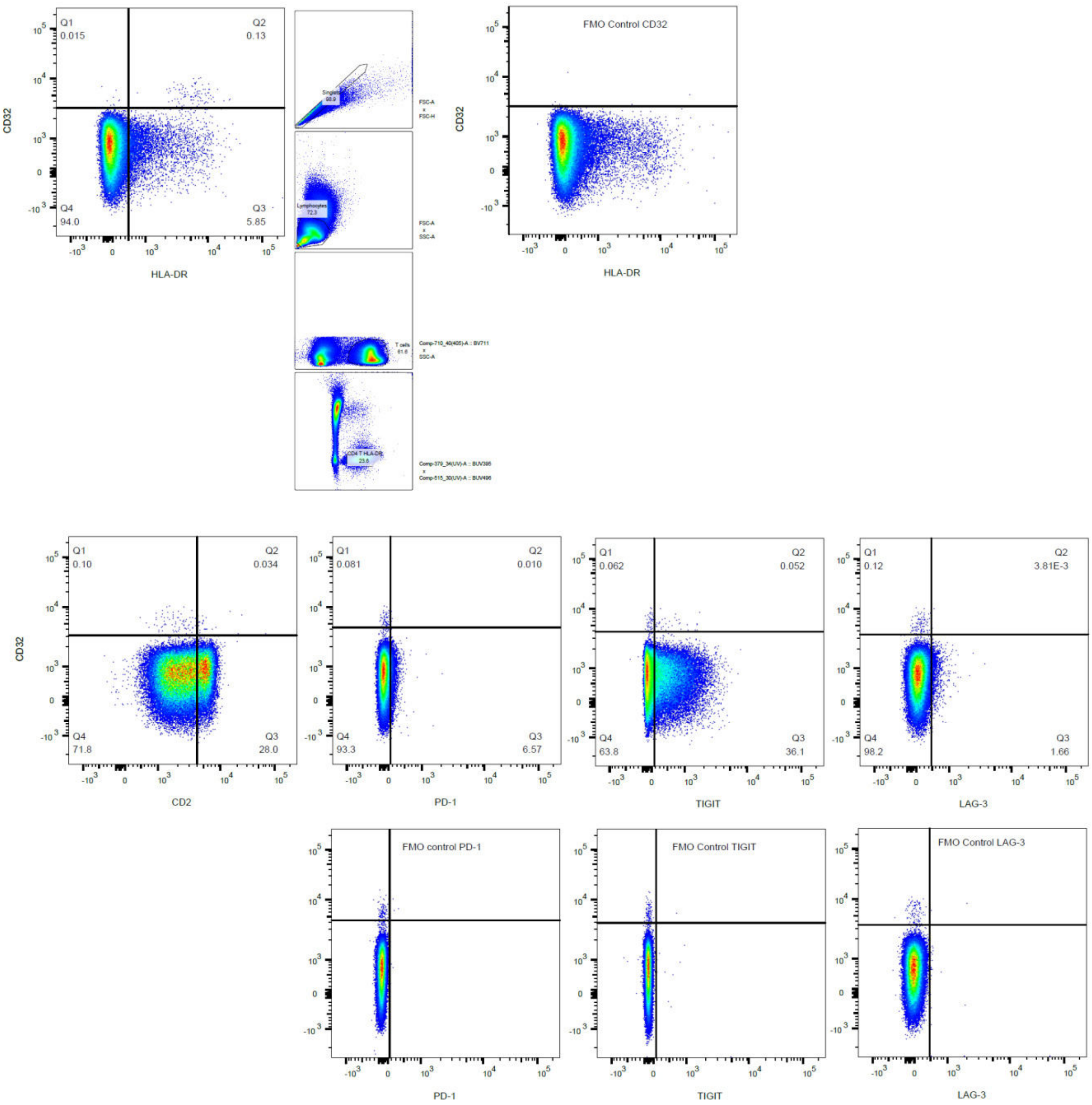

Figure S1. Representative example of the gating strategy. Fluorescence Minus One (FMO) controls were used for all samples. FMOs for CD32, as well as PD-1, TIGIT, and LAG-3 are presented. FSC = forward scatter, and SSC = side scatter. Related to Figures 1-3.

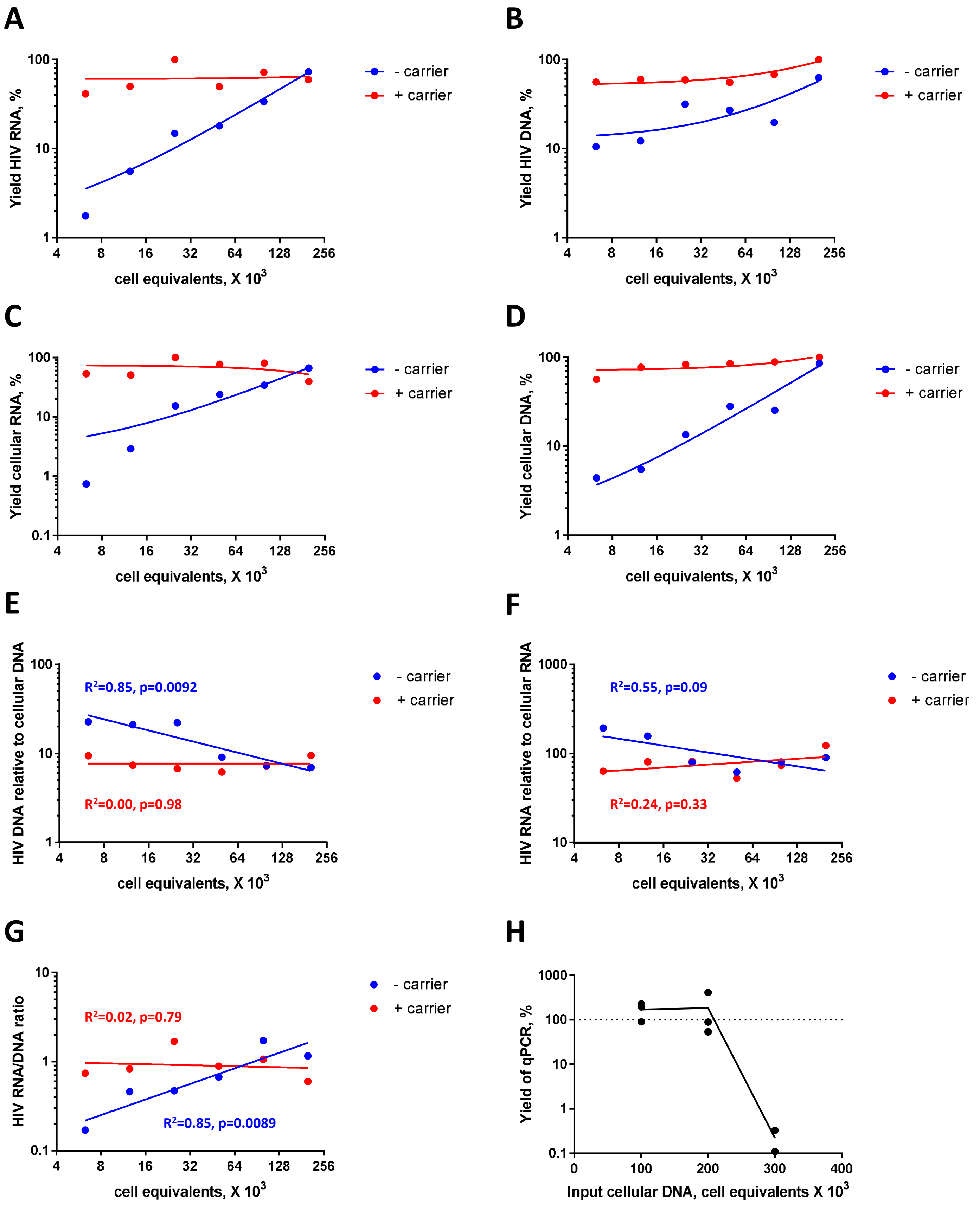

Figure S2. (A-G) Effect of poly-A carrier RNA on the yield of nucleic acid extraction. (A) HIV RNA yield, (B) HIV DNA yield, (C) cellular RNA yield, (D) cellular DNA yield, (E) HIV DNA relative to cellular DNA, (F) HIV RNA relative to cellular RNA, (G) HIV RNA/DNA ratio, with and without the addition of carrier RNA. R2 and p values in (E-G) were calculated by linear regression on log-transformed values. (H) Effect of cellular DNA on the yield of HIV qPCR. Increasing amounts of cellular DNA isolated from uninfected donor PBMCs were spiked in triplicate into the HIV DNA quantitation standard plasmid prior to qPCR. Shown is the yield of qPCR as a percentage of the mean output without the spiked DNA. Related to Figures 1, 3-6.

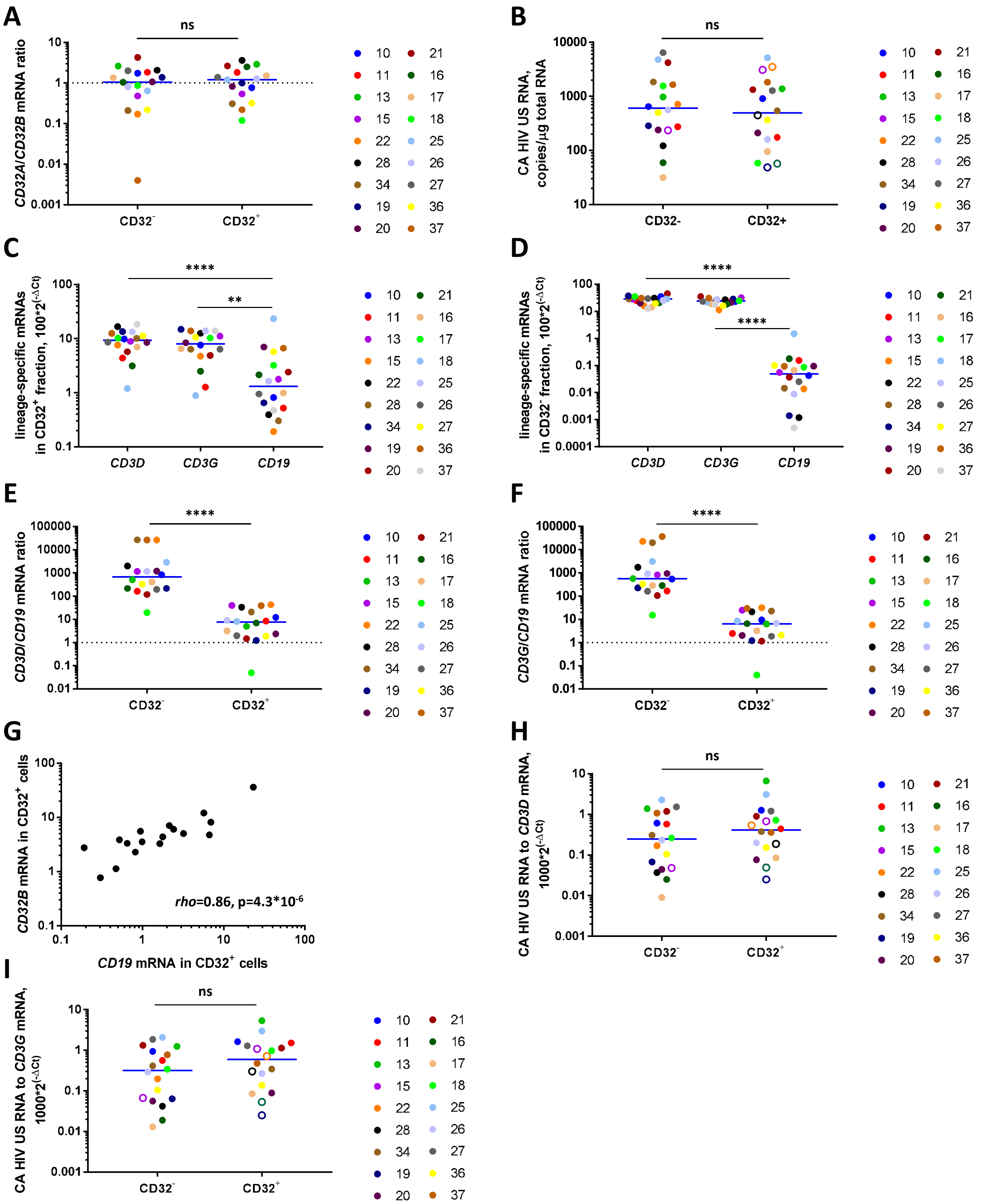

Figure S3. (A) CD32A/CD32B mRNA ratio in CD32+ and CD32- fractions of CD4+ T cells. (B) Cell-associated (CA) unspliced (US) HIV RNA levels, normalized to the total cellular RNA (by 18S ribosomal RNA), in CD32+ and CD32- fractions. (C, D) Lineage-specific mRNA levels in CD32+ (C) and CD32- (D) fractions. (E) CD3D/CD19 mRNA ratios in CD32+ and CD32- fractions. (F) CD3G/CD19 mRNA ratios in CD32+ and CD32- fractions. (G) Correlation between CD19 and CD32B mRNA levels in the CD32+ fraction. All host mRNA levels were normalized to GAPDH mRNA. (H, I) CA US HIV RNA levels, normalized to CD3D (H) and CD3G (I) mRNA levels, in CD32+ and CD32- fractions. Open circles depict undetectable values, censored to the assay detection limits. The latter depended on the amounts of input cellular RNA and therefore differed between samples. Wilcoxon tests (panels A, B, E, F, H, I), Friedman tests with Dunn's post-tests (panels C, D), or Spearman tests (panel G) were used to calculate statistical significance. ****, $p<0.0001$; **, $0.001<p<0.01$; ns, not significant. Related to Figure 4.

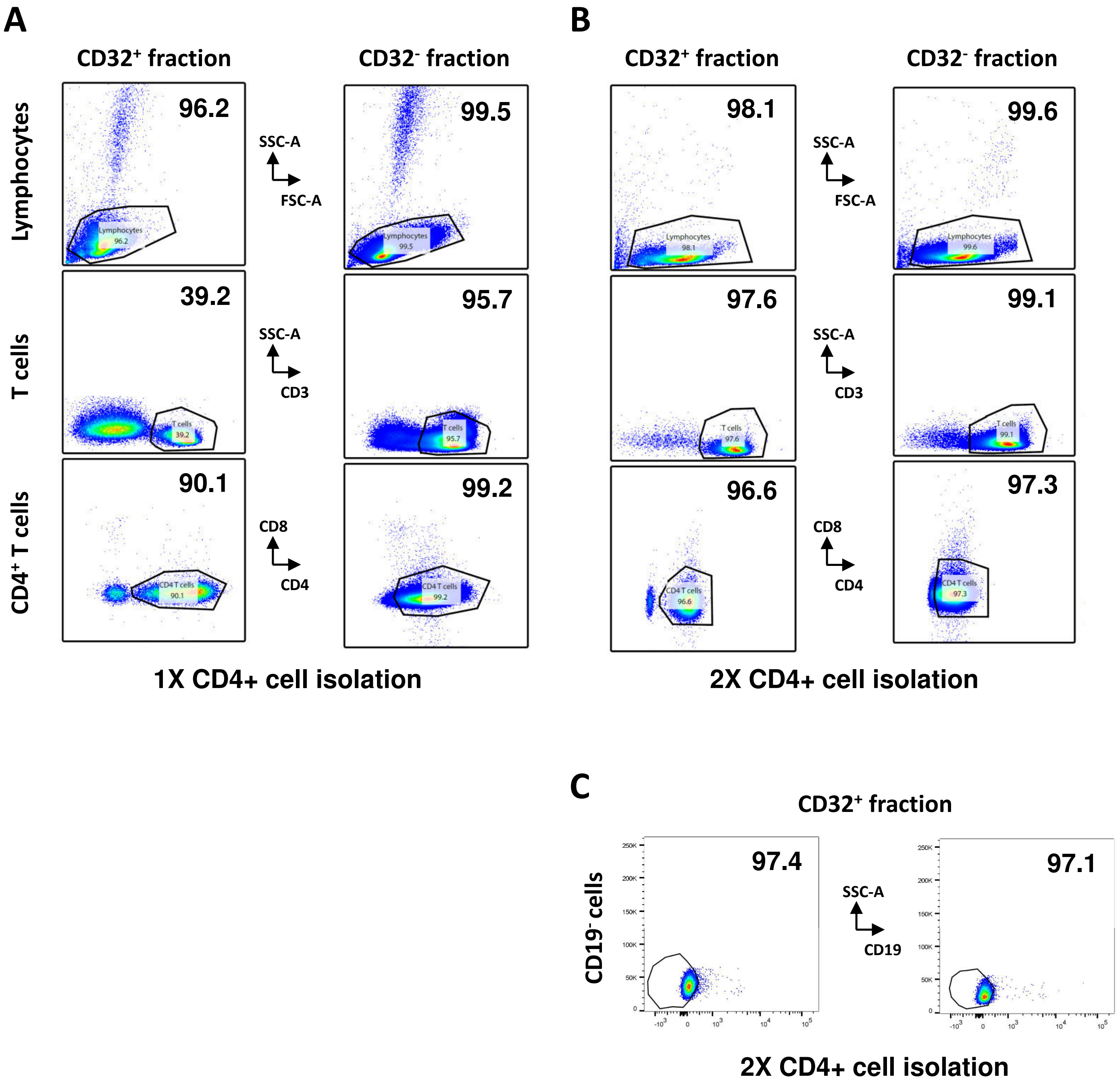

Figure S4. (A, B) Representative examples of the purity of the lymphocyte (among total PBMC), T-cell (among total lymphocytes) and CD4+ T-cell (among total T cells) populations following the isolation of CD4+ T cells by magnetic sorting. Panel A: CD4+ T-cell isolation has been performed once. Panel B: CD4+ T-cell isolation has been performed twice. FACS analyses are presented for the CD32+ and CD32- populations. Percentages of the desired cell populations are shown on the figures. FSC, forward scatter. SSC, side scatter. (C) CD32+CD4+ T cells isolated by two rounds of magnetic sorting are largely free from B-cell contamination. CD19 stainings are shown for two participants (94 and 95, Table S1). Percentages of CD19- cells are shown. Related to Figures 4 and 5.

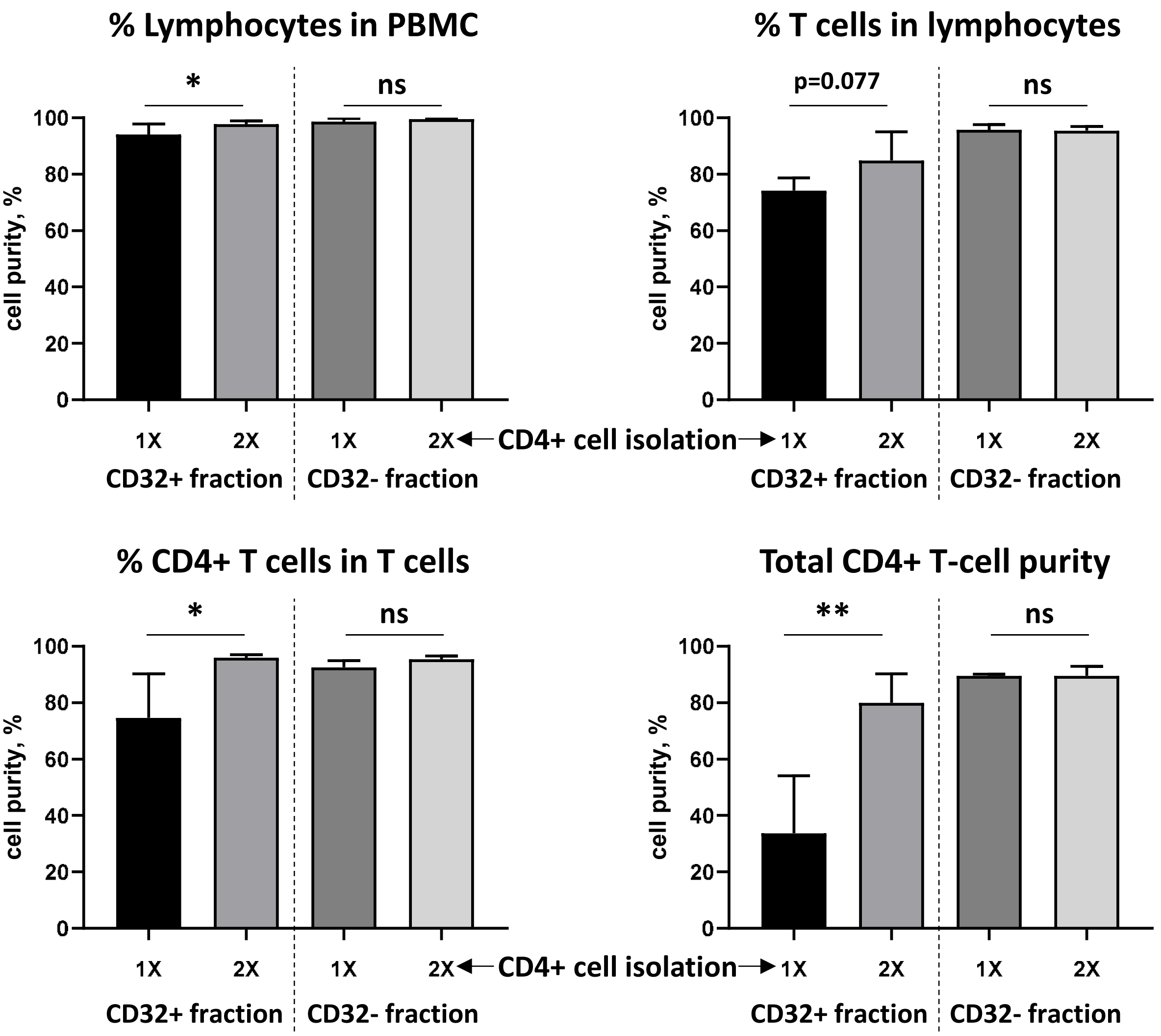

Figure S5. Purity of the same cell populations as in Figures S4A-B, plus total CD4+ T-cell purity, was compared between one (n=3; participants 31, 32, and 33) and two (n=10; participants 45, 46, 47, 77, 79, 80, 81, 82, 83, and 84) consecutive rounds of CD4+ T-cell isolation, followed by one round of CD32+ cell isolation. Comparisons are presented separately for CD32+ and CD32- fractions. Unpaired t-tests were used to calculate statistical significance. **, $0.001<p<0.01$; *, $0.01<p<0.05$; ns, not significant. Related to Figures 4 and 5.

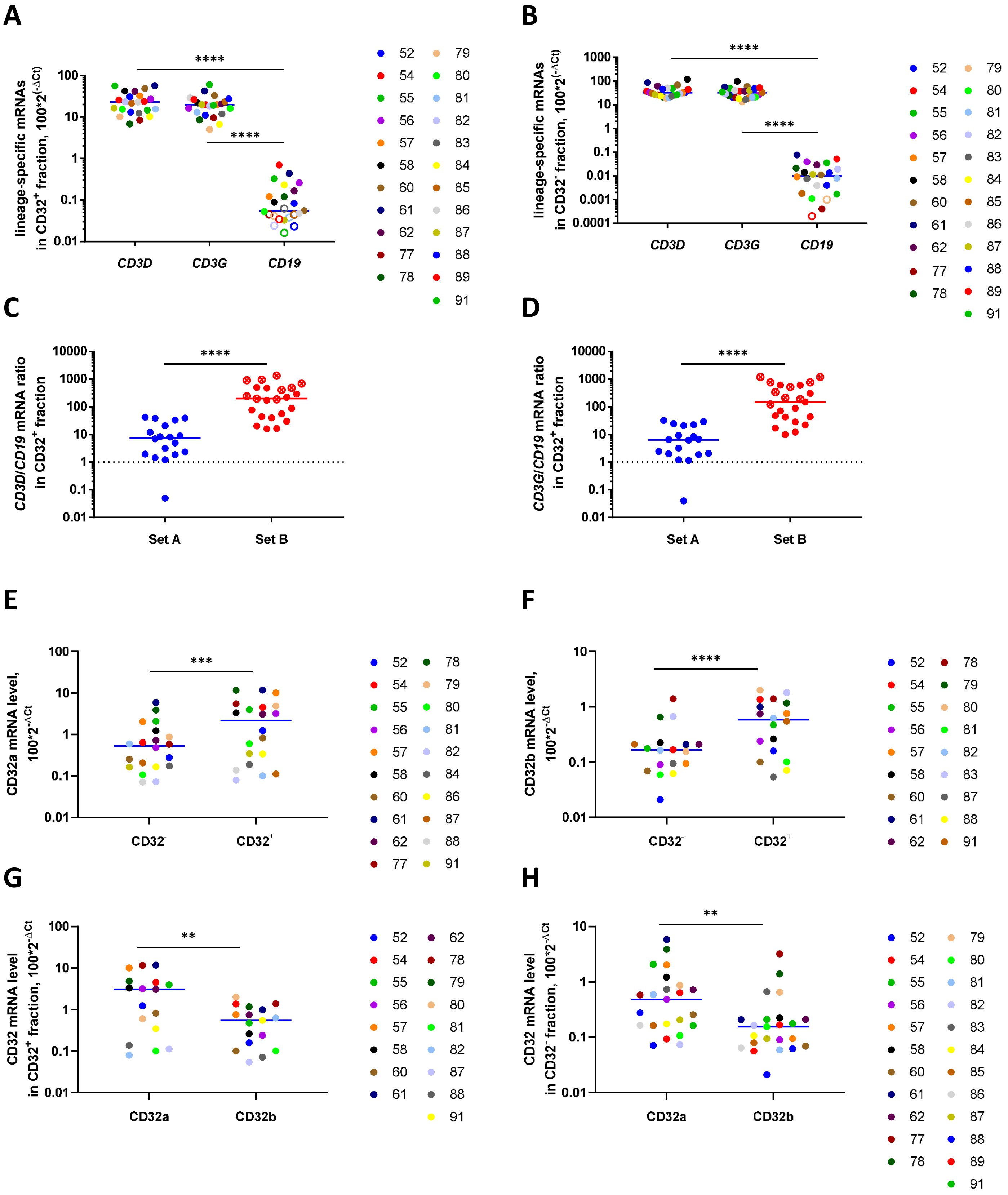

Figure S6. (A, B) Lineage-specific mRNA levels in CD32+ (A) and CD32- (B) fractions. Open circles depict undetectable values, censored to the detection limits. The latter depended on the amounts of GAPDH mRNA and therefore differed between samples. (C, D) Comparison between CD3D/CD19 (C) and CD3G/CD19 (D) mRNA ratios in the CD32+ fraction between patient sets A and B. Open crossed circles correspond to the samples where CD19 mRNA values were undetectable and censored to the detection limits; because all CD3D and CD3G mRNA values were detectable, these circles depict the lower limits of CD3D/CD19 or CD3G/CD19 mRNA ratios. (E, F) Comparison of CD32A (E) and CD32B (F) mRNA levels between CD32+ and CD32- fractions of CD4+ T cells in Set B (only pairs where both values were detectable are shown). (G, H) Comparison between CD32A and CD32B mRNA levels in CD32+ (G) and CD32- (H) fractions of CD4+ T cells in Set B (only pairs where both values were detectable are shown). All mRNA levels were normalized to GAPDH mRNA. Friedman tests with Dunn's post-tests (panels A, B), Mann-Whitney tests (panels C, D), or Wilcoxon tests (panels E-H) were used to calculate statistical significance. ****, $p<0.0001$; ***, $0.0001<p<0.001$; **, $0.001<p<0.01$. Related to Figure 5.

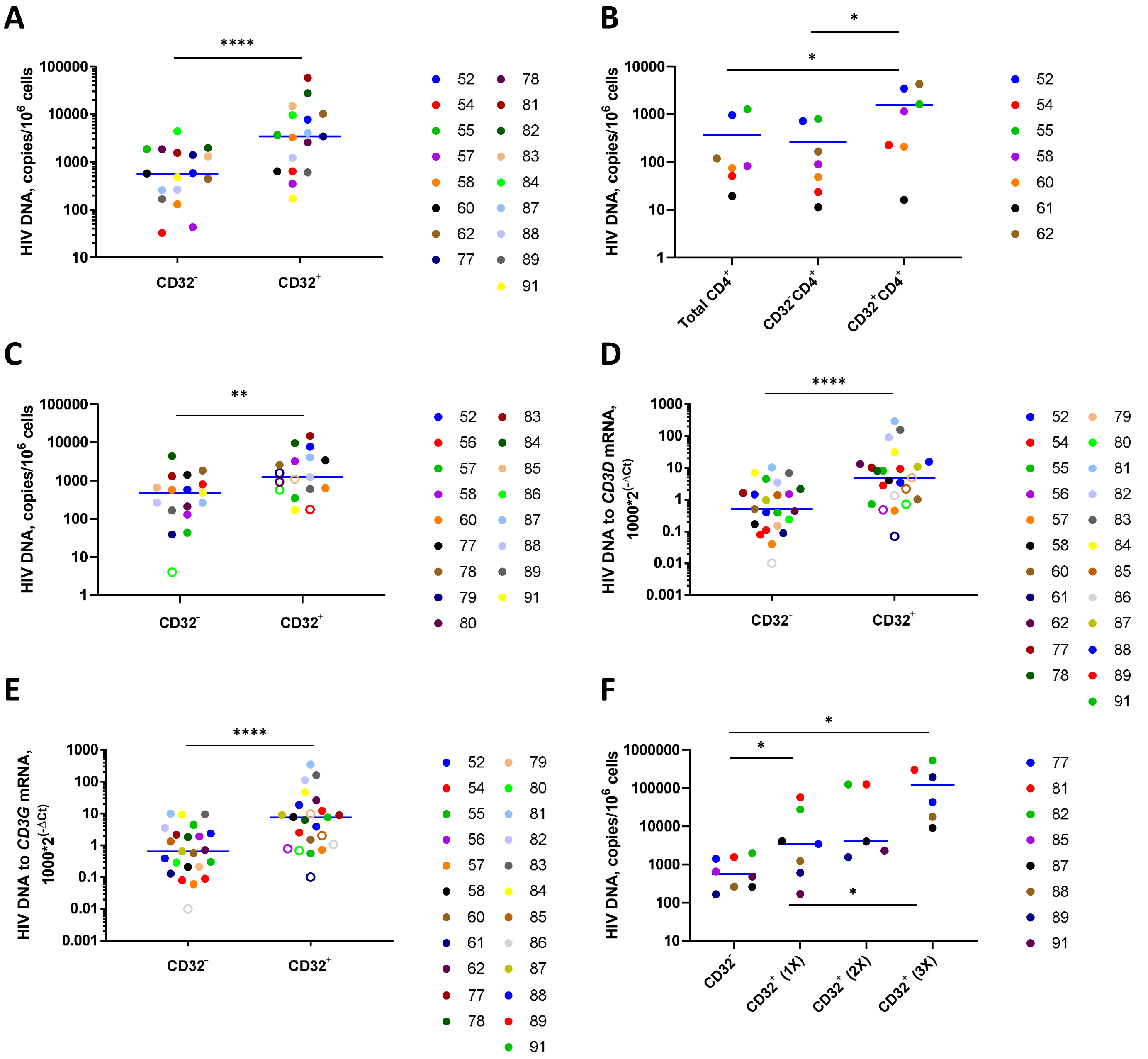

Figure S7. (A) Comparison of HIV DNA levels between CD32+ and CD32- fractions of CD4+ cells in a subset of Set B individuals with both HIV DNA values detectable in every pair (n=17). (B) Comparison of HIV DNA levels between total CD4+, CD32-CD4+, and CD32+CD4+ cell fractions in a subset of Set B individuals (n=7). (C) Comparison of HIV DNA levels between CD32+ and CD32- fractions of CD4+ cells in a subset of Set B individuals with ≥4 years of suppressive ART (n=17). (D, E) Comparison of HIV DNA levels in Set B between CD32+ and CD32- fractions, when HIV DNA was normalized to CD3D (D) and CD3G (E) mRNAs. Open circles depict undetectable values, censored to the assay detection limits. The latter depended on the amounts of CD3 mRNA and therefore differed between samples. (F) Comparison of HIV DNA levels between the CD32- fraction and the CD32+ fraction obtained after one, two, or three consecutive rounds of CD32+ positive selection. Only detectable values are shown. Wilcoxon tests were used to calculate statistical significance. ****, $p<0.0001$; **, $0.001<p<0.01$; *, $0.01<p<0.05$. Related to Figures 5 and 6.

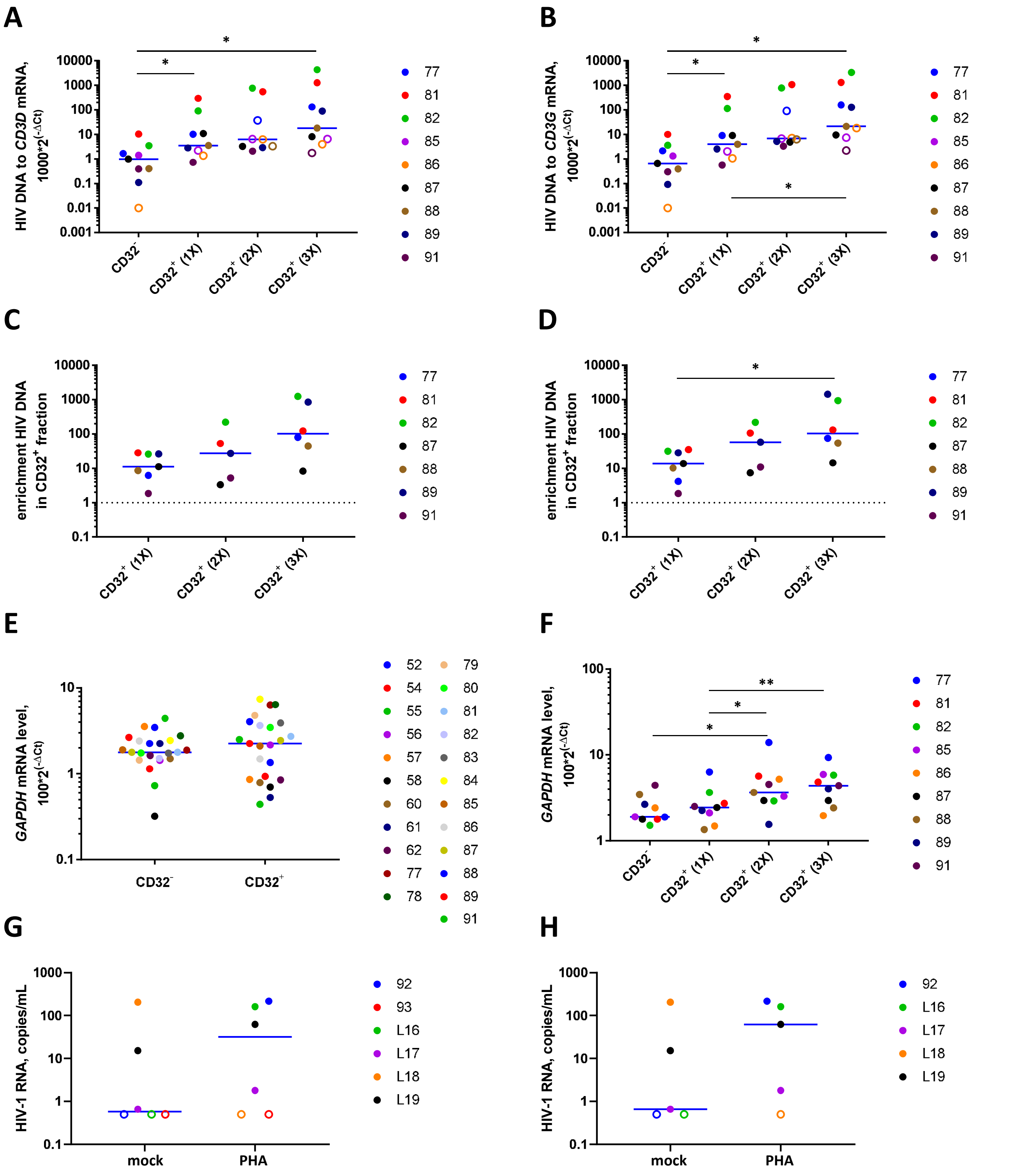

Figure S8. (A, B) Comparison of HIV DNA levels, normalized to the CD3D (A) and CD3G (B) mRNA levels, between the CD32- fraction and the CD32+ fraction obtained after one, two, or three consecutive rounds of CD32+ positive selection. (C, D) Comparison of the enrichment for HIV DNA (normalized to CD3D (C) or CD3G (D) mRNA) between the CD32+ fractions obtained after one, two, or three consecutive rounds of CD32+ positive selection. Open circles depict undetectable values, censored to the assay detection limits. The latter depended on the amounts of CD3 mRNA and therefore differed between samples. (E) Comparison of GAPDH mRNA levels, normalized to the total cellular RNA (by 18S ribosomal RNA), between the CD32- and the CD32+ fractions in Set B. (F) Comparison of GAPDH mRNA levels, normalized to the total cellular RNA (by 18S ribosomal RNA), between the CD32- fraction and the CD32+ fraction obtained after one, two, or three consecutive rounds of CD32+ positive selection. Wilcoxon tests were used to calculate statistical significance. **, $0.001<p<0.01$; *, $0.01<p<0.05$. (G, H) Levels of extracellular HIV virion RNA in mock and PHA-stimulated cultures in the ex vivo HIV reactivation assay: (G) all participants (n=6), (H) participants (n=5) included in the calculation of fold induction (Figure 7C). The units of measurement are copies per mL of culture supernatant. Median values are shown. Open circles depict undetectable values, censored to the assay detection limits. Related to Figures 6 and 7.
